# Quantifying Regional and Age-Dependent Microstructural Changes in Porcine Ventricles from Neonatal to Adulthood Using DT-MRI and TPEF-SHG Microscopy

**DOI:** 10.1007/s10439-026-04143-4

**Published:** 2026-04-13

**Authors:** Faizan Ahmad, James Paul Barnett, Ali Bienemann, Carol-Ann Janes, Peter Theobald

**Affiliations:** 1 Dept. of Life and Sports Sciences, Birmingham City University, City South Campus, Westbourne Road, Birmingham, B15 3TN UK; 2 Bristol Medical School, Translational Health Studies (THS), University of Bristol, Bristol, BS8 1QU UK; 3 School of Engineering, Cardiff University, Queen’s Buildings - The Parade, Newport Road, Cardiff, CF24 3AA UK

**Keywords:** Heart ageing, Cardiomyocyte ageing, Collagen fibrils ageing, Cardiomyocytes rotation, Collagen fibrils rotation, Cardiomyocytes dispersion, Collagen fibrils dispersion, Magnetic resonance imaging, Two-photon excitation microscopy, Second-harmonic generation microscopy

## Abstract

The heart undergoes substantial structural changes in response to new physiological demands, which occur with the rapid opening of pulmonary circulation immediately after birth. The dependence on pulmonary circulation causes an immediate increase in ventricular workload, resulting in microstructural changes that serve to maintain overall physiological homeostasis. Ageing continues to evolve the heart’s structure due to increased myocardial tissue stress and strain, initiating the formation of a new extracellular matrix to facilitate the physiology of an adult. Quantifying the region-specific and age-dependent microstructural changes in tissue due to ageing is pivotal for the development of constitutive models for computational simulations. This study aimed to determine the microstructure of porcine ventricles at four time points from neonatal to adulthood. The three-dimensional microstructure was investigated using diffusion-tensor magnetic resonance imaging, two-photon excited fluorescence and second-harmonic generation microscopy to quantify fibre tractography, fractional anisotropy (FA), spherical measure, rotation and dispersion of cardiomyocytes and collagen fibrils. The results revealed that the left ventricle possessed greater FA than the right. Adult hearts demonstrated smaller FA than the young. The anterior left and right ventricles exhibited greater cardiomyocyte and collagen fibril rotation and dispersion than the posterior. The adult hearts possessed greater cardiomyocyte and collagen fibril rotation and dispersion than young hearts. The right ventricle demonstrated greater cardiomyocyte rotation in the younger hearts, and the Left in the adult. This study provides baseline data that should prove useful to bioengineers, researchers, and mathematicians in developing region-specific and age-dependent constitutive models to enhance the accuracy and bio-fidelity of computational simulations.

**Supplementary Information:**

The online version contains supplementary material available at https://doi.org/10.1007/s10439-026-04143-4.

## Introduction

The heart undergoes substantial structural changes to adapt to the new physiological demands immediately after birth. This involves the immediate opening of the pulmonary circulation, with rapid closure of the patent ductus arteriosus and the foramen ovale, which bypasses blood from the right atrium/pulmonary artery to the left atrium/aorta [1–3]. The dependence on pulmonary circulation results in immediate microstructural changes in the heart, including cardiomyocyte hypertrophy, hyperplasia, and extracellular collagen deposition [4, 5]. Within the first 14 days, the porcine left ventricle increases in mass 4.5-fold, cell volume increases 3.5-fold, whilst cellular proliferation increases 2-fold [5]. The increase in interstitial collagen deposition also occurs, peaking 15-20 days postnatal [6]. Ageing gradually evolves the heart’s structure due to increased stress and strain in the myocardial tissue, initiating the formation of a new extracellular matrix (ECM) to facilitate the functional physiology of an adult [7, 8]. These physiological changes occur to support the ventricular wall functionality, which differs between neonates and adults, as the former can only increase cardiac output by increasing heart rate (although only to a limited extent), whereas the adult heart can also increase stroke volume [9, 10]. Significant microstructural changes have been reported in the porcine ventricles during the transition from neonatal to adulthood [5, 11, 12]. This includes an increase in the proportion of contractile versus fibrous elements, collagen fibril density, and mono-and bi-nucleated cell concentrations [11–14]. Ageing also predominantly increases the collagen fibril crosslinking and assembly in the ventricles [15].

The ventricular myocardium has an intricate cellular structure. The extracellular matrix (ECM) comprises collagen types I and III, elastin, proteoglycans and glycoproteins, within which are arranged cardiomyocytes, fibroblasts, endothelial and smooth muscle cells. This highly organised ventricular myocardium structure contributes to maintaining the coordinated contraction of the heart [16]. The three-dimensional ECM structure includes collagen fibres and cardiomyocytes, both of which significantly contribute to gross biomechanical properties during the diastolic and systolic phases. Cardiomyocytes are the fundamental contractile units of the heart, arranged parallel to the epicardial surface in a multi-layered circumferential helix, the precise rotation and dispersion of which varies across the heart and through the epicardium to the endocardium (i.e. through the wall thickness) [17, 18]. These structural variations are known to provide the myocardium with longitudinal and circumferential strength, the two directions that endure the highest stress during the cardiac cycle [19]. The region-specific variations in cardiomyocyte structure provide the pathway for generating ventricular torsion and maintaining the effective ejection fraction of the left ventricle [20]. It also contributes significantly to the spatiotemporal patterns of mechanical activation in different layers and regions of the ventricular wall [21]. Neither the cardiomyocytes nor the collagen fibres are perfectly aligned in the ventricular myocardium, with their ‘fibre orientation’, ‘fibre angle’, ‘fibre rotation’, and ‘angular dispersion’ (i.e. disorganisation) essential to maintaining myocardial stiffness and anisotropy during the cardiac cycle [22]. The fibre rotation and dispersion also influence the passive and active behaviour of myocardial tissue [23–25].

Computational simulations of the heart provide a non-invasive and controllable platform for understanding, predicting, and managing the effects of cardiac diseases [26–31]. The critical need to develop structure-based constitutive models during cardiac growth and remodelling (ageing) to investigate the interaction between structural remodelling and hypertrophy in the right ventricle in response to pulmonary arterial hypertension has been identified in the literature [32, 33]. The strong correlation between fibre architecture and impaired contractility during heart failure with preserved ejection fraction has also been reported [34]. Hence, the significance of incorporating image-based microstructural data, including the 3D micro-anatomical arrangement of cardiomyocytes and collagen fibrils, in computational models for the study of cardiac disease and patient-specific treatments has been emphasised in the literature [35, 36]. Computational simulations have been established to predict and explore the impact of interventions on the microstructural, biomechanical, and electrophysiological aspects of the heart [25, 26, 30, 37–39]. Constitutive models are used to estimate the material parameters for these computational simulations. These constitutive models use microstructural data of myocardial tissue, including fibre rotation and dispersion, for computational simulations [40–46]. The computational simulation accuracy is dependent on the ability to simulate biological tissue behaviour accurately. At its most simplistic, inputting the single-region microstructural data, applied ‘globally’ to the entire heart, enables simulations, though with limited accuracy. Conversely, tissue structure mapped across different regions, including anterior and posterior aspects of the left and right ventricles, describes multiple strain states and modes, providing a true representation of the tissue’s region-specific and age-dependent behaviour. Currently, a lack of region-specific and age-dependent microstructural data to describe heart tissue ageing from neonatal to adult stages limits the widespread use and bio-fidelity of sophisticated constitutive models for performing accurate computational simulations of heart ageing, to investigate the impact of disease progression and interventions on cardiac function.

The current understanding of cardiac tissue microstructure does not account for region-specific, age-dependent macro- and microscopic structural variations from the neonatal to the adult stage. The data are fragmented in the literature, reporting microstructural data obtained using different experimental techniques and tissues harvested from various mammalian hearts, with different regions, genders, and ages [47–55]. This prevents the development of a continuum of region-specific and age-dependent microstructural data spanning birth to death, limiting the potential of computational modelling for accurately simulating the region-specific and age-dependent behaviour of the heart. Various three-dimensional imaging methodologies have been used to analyse the microstructure of myocardial tissue, including magnetic resonance microscopy, micro-computed tomography, ultrasound techniques, and optical coherence tomography [56]. All these imaging modalities provide detailed information on the microstructural organisation of cardiomyocytes. Two-photon excited fluorescence (TPEF), second-harmonic generation (SHG), and confocal microscopy imaging can quantify both the cardiomyocyte and collagen fibril microstructure in myocardial tissue and have been used in previous studies [57, 58]. TPEF and SHG enable the optical sectioning of relatively thick tissue samples. The former can image elastin and cardiomyocytes by exciting endogenous fluorophores, whilst SHG provides a deeper insight into those molecules lacking a centre of symmetry (e.g., collagen, microtubules, and myosin) [59, 60]. When used in tandem, the two techniques provide a microscopic, 3D representation of the interplay between key proteins, resulting in the determination of cardiomyocyte and collagen fibril rotation and dispersion parameters in thicker tissue samples [61]. Diffusion tensor magnetic resonance imaging (DT-MRI) provides a platform for assessing the structural characteristics of the heart, such as fractional anisotropy (FA), to quantify water diffusion anisotropy for describing the directional coherence of cardiomyocyte orientation [62, 63]. DT-MRI can also be used to evaluate fibre-tractography and spherical measurements (i.e., roundness of the geometry) to demonstrate the gross structural and geometrical changes in the heart due to ageing.

This study used DT-MRI and TPEF-SHG to characterise the macro-and microstructural parameters of cardiac porcine tissue, focusing on the potentially different regional structural variations in the anterior and posterior aspects of the left and right ventricles ‘free-walls’. A porcine model, which is a widely accepted surrogate animal model in cardiac-related research [64–66], was used throughout, with tissue harvested from animals of four age groups, to broadly capture early, mid and later stages of life.

## Materials and Methods

### Materials

**Fig. 1:**
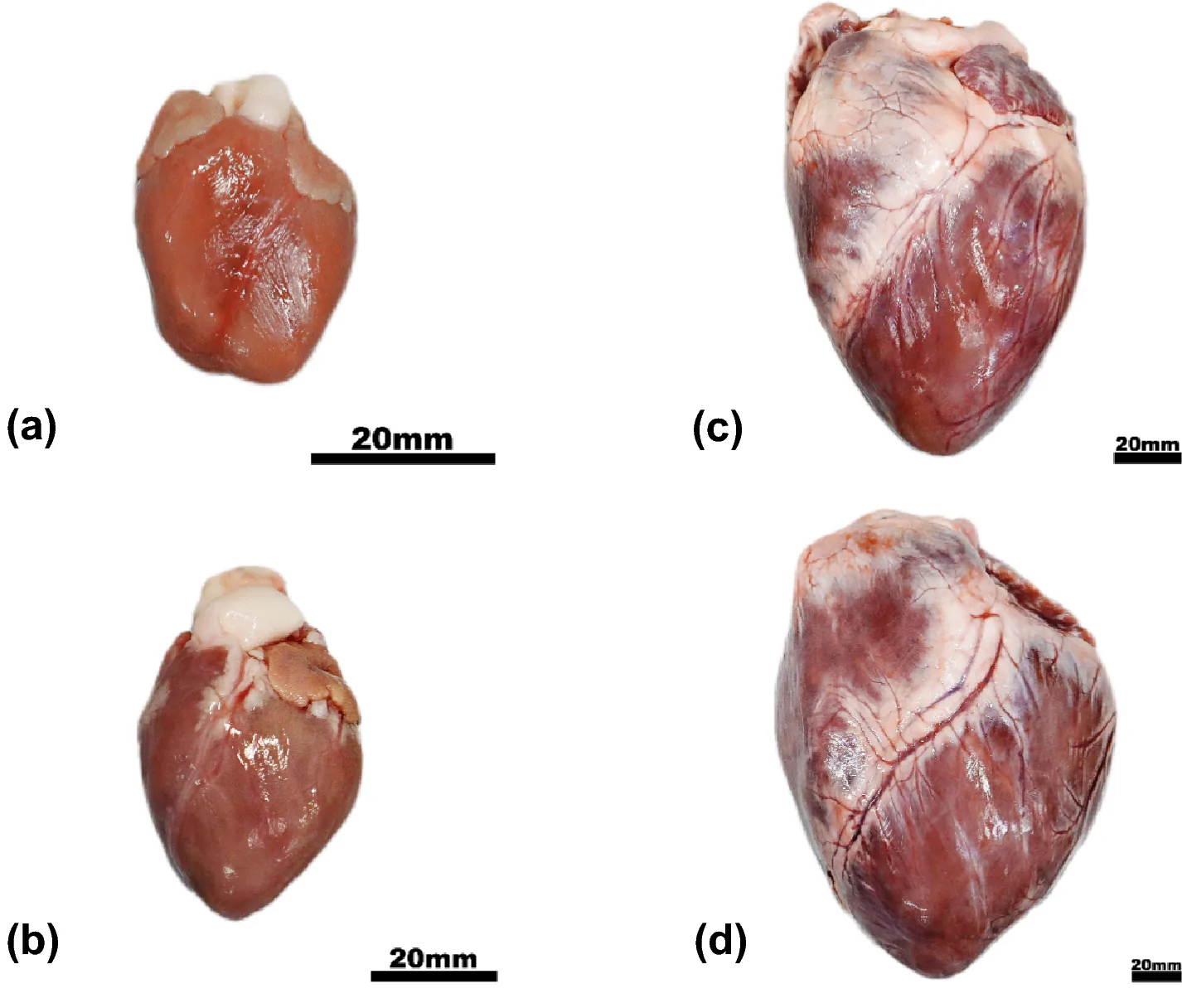
Harvested porcine hearts of four age groups, namely 7-day-old (**a**), 14-day-old (**b**), 7 months old (**c**), and 3 years old (**d**). Scale bar = 20 mm

**Fig. 2:**
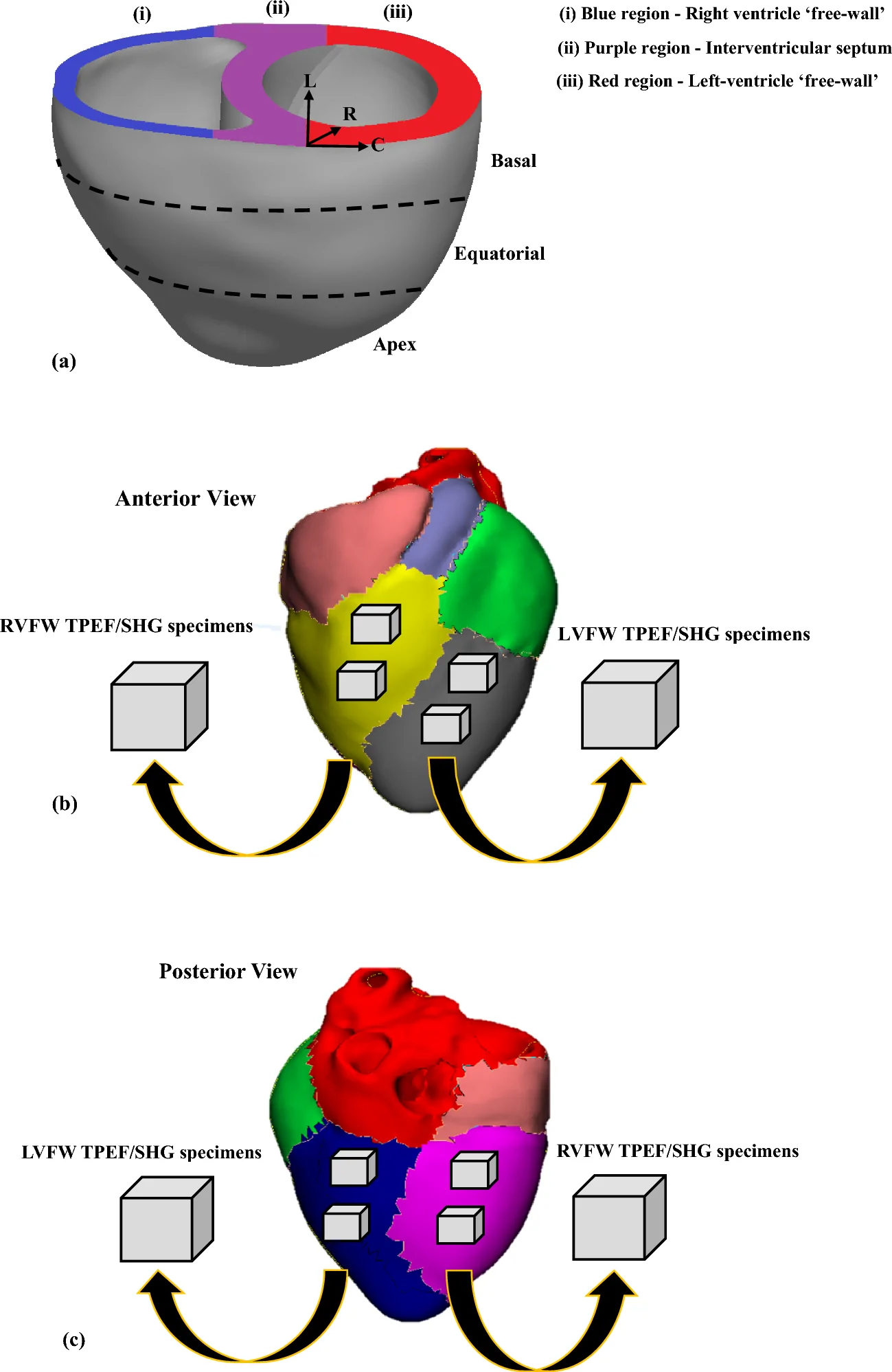

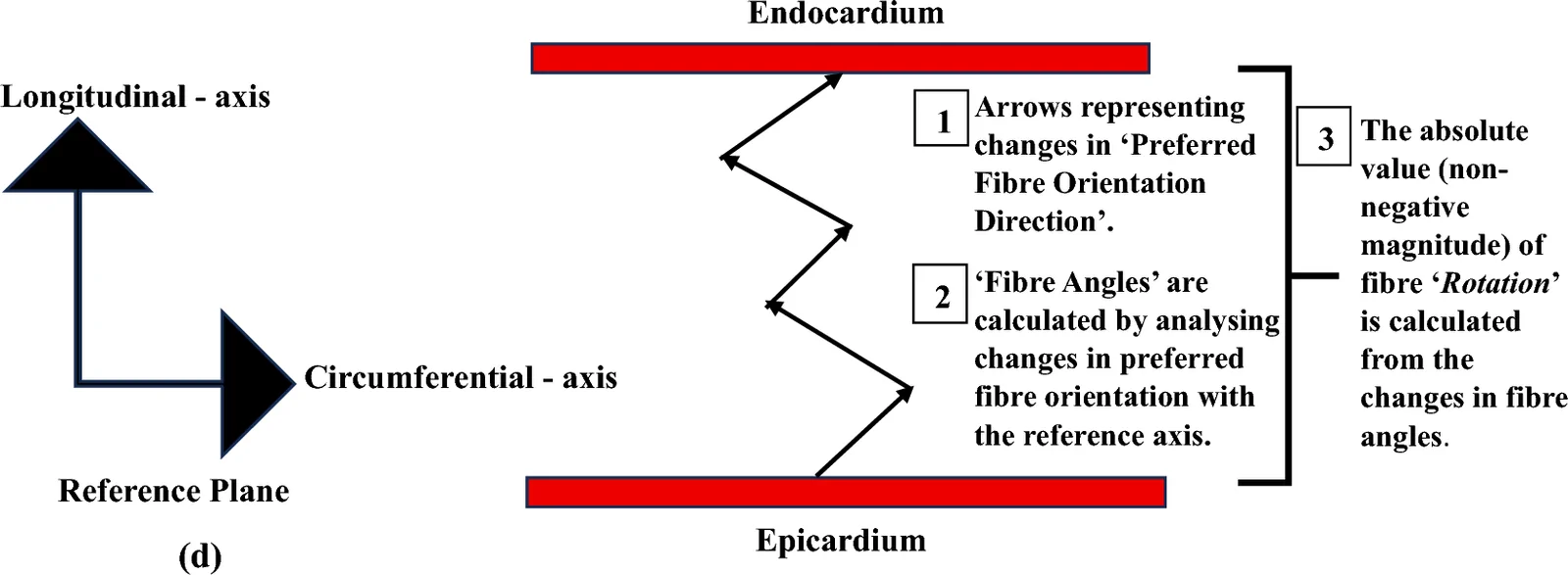
**a** 3D schematic model of the porcine ventricular myocardium composed of the right ventricular free wall (RVFW), highlighted with the blue region; the interventricular septum, highlighted with the purple region; and the left ventricular free wall (LVFW), highlighted with the red region. The circumferential-radial-longitudinal (CRL) coordinate system is defined to describe the principal axes of the heart. The longitudinal axis is defined vertically from the centre of the base to the apex. The circumferential axis is perpendicular to both the longitudinal and radial directions and is oriented in a counterclockwise direction around the short axis. The radial axis is directed perpendicular to the epicardial surface, pointing away from the ventricular epicardium (i.e., through thickness). **b** Squared specimens (15x15 mm) were dissected from the anterior aspect of the basal and equatorial regions of the LVFW (grey region) and RVFW (yellow region) to perform TPEF/SHG microscopic imaging through the absolute thickness (i.e., epicardium to endocardium). **c** Squared specimens (15x15 mm) were dissected from the posterior aspect of the basal and equatorial regions of the LVFW (blue region) and RVFW (pink region) to perform TPEF/SHG microscopic imaging through the absolute thickness (i.e., epicardium to endocardium). **d** Schematic diagram to demonstrate the methodology of calculating fibre angles and fibre rotation from the preferred fibre orientation direction from epicardium to endocardium with reference to the circumferential – longitudinal reference plane axis in three steps. This schematic diagram is not scaled. (For interpretation of the references to colour in this figure legend, the reader is referred to the web version of this article)

**Fig. 3:**
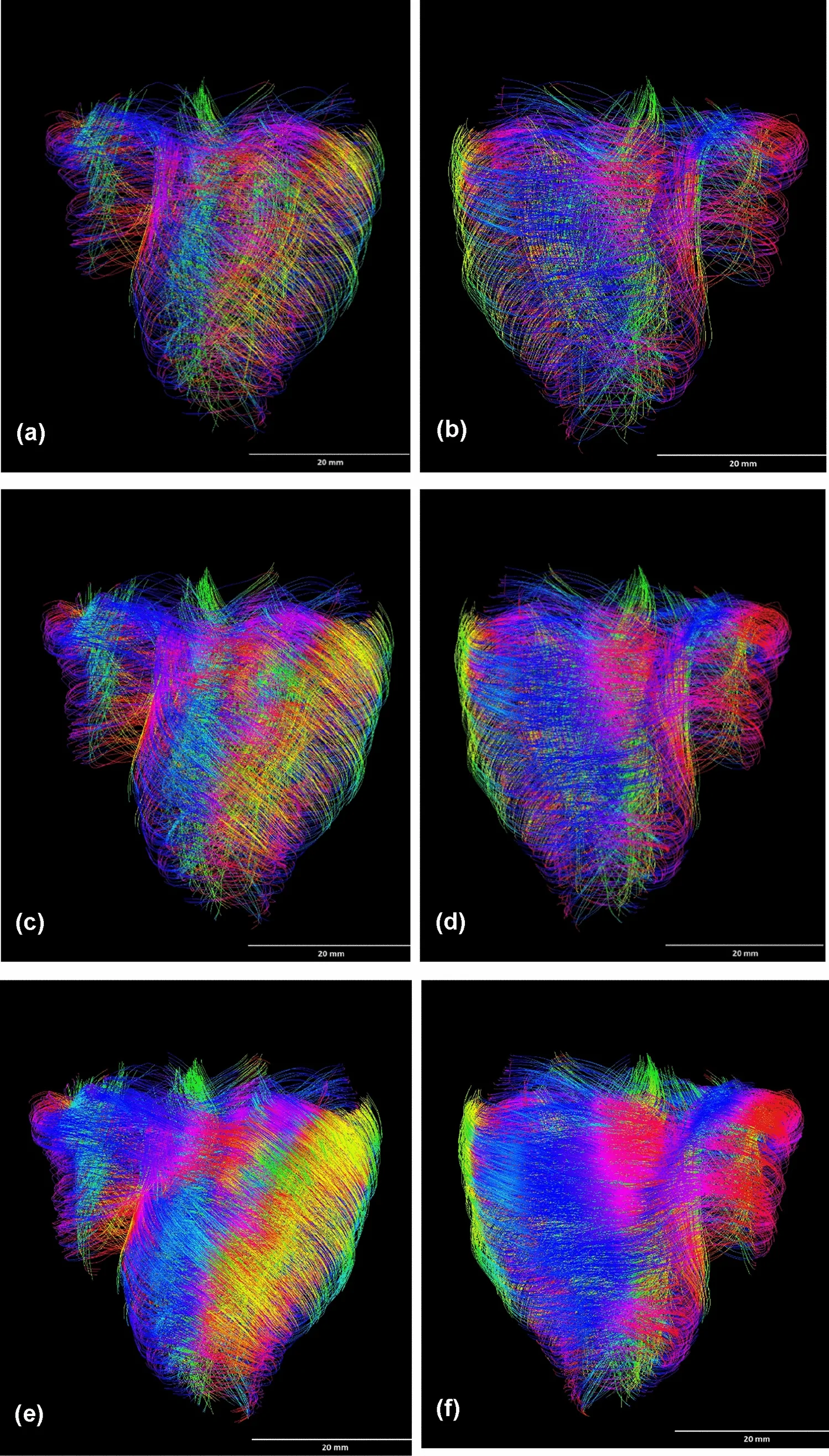

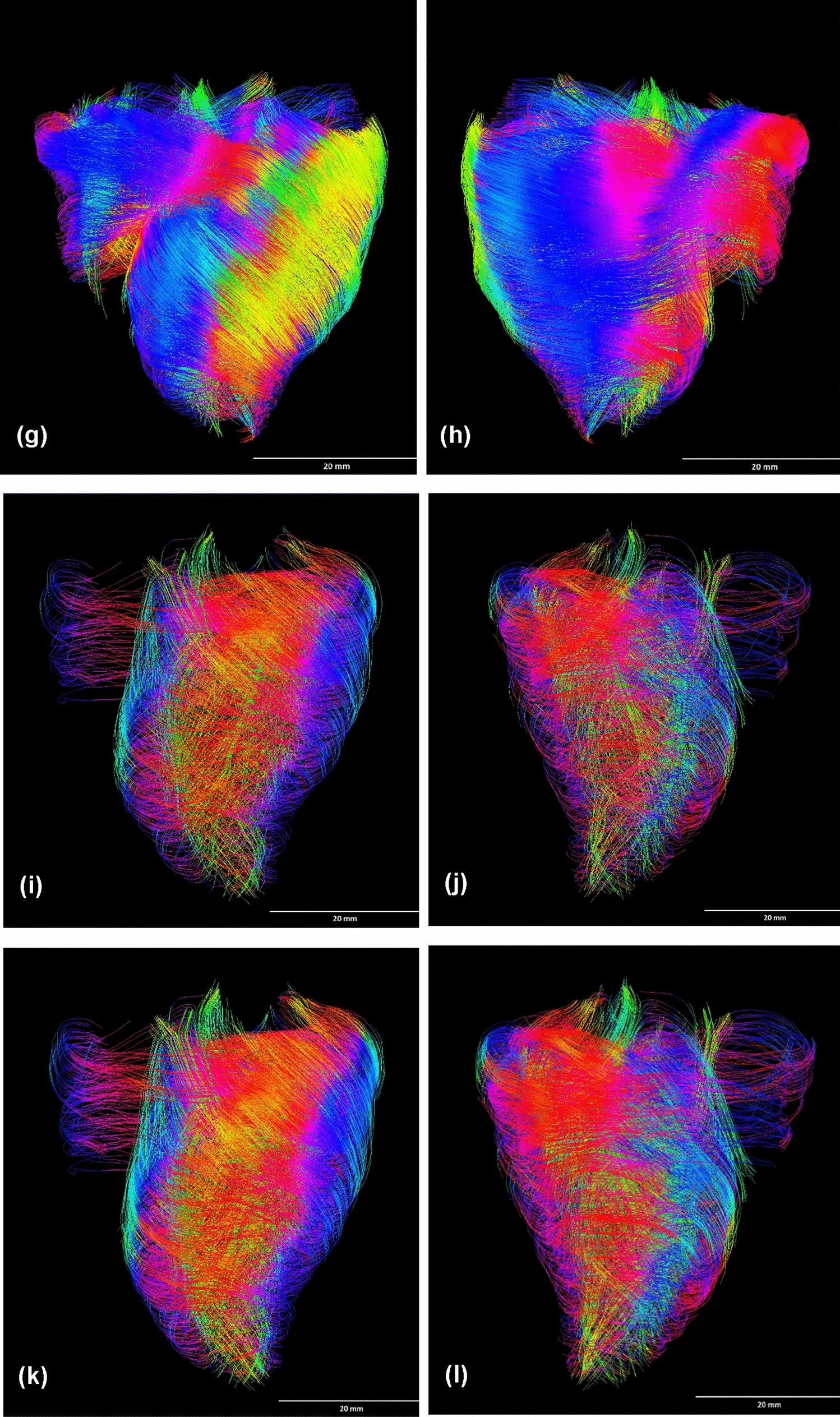

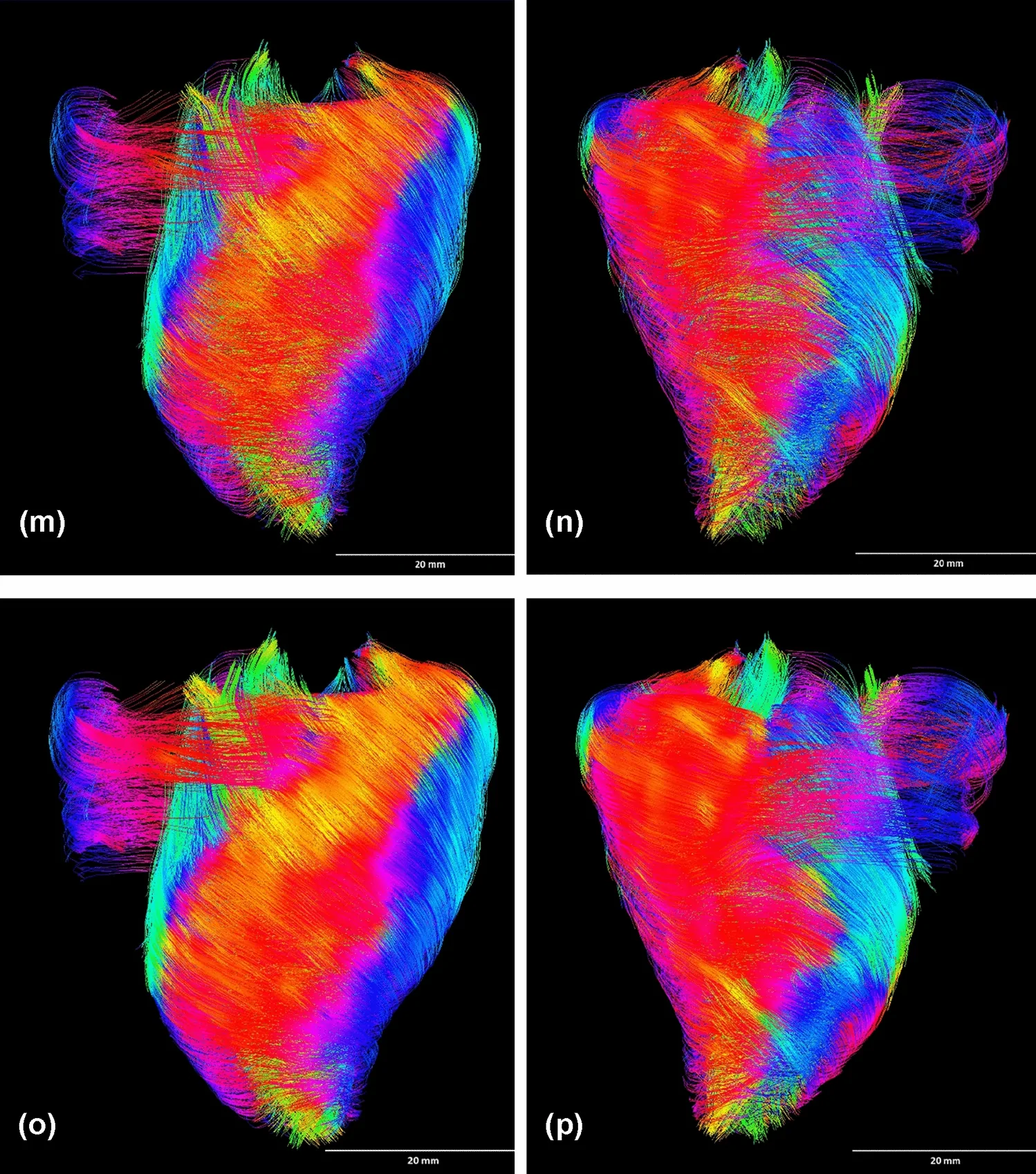
Post-processed DT-MRIs of a *7-day-old* porcine heart exhibiting 15% fibre density of anterior (**a**) and posterior (**b**) aspects, 25% fibre density of anterior (**c**) and posterior (**d**) aspects, 50% fibre density of anterior (**e**) and posterior (**f**) aspects, and 100% fibre density of anterior (**g**) and posterior (**h**) aspects. Post-processed DT-MRIs of a *14-day-old* porcine heart exhibiting 15% fibre density of anterior (**i**) and posterior (**j**) aspects, 25% fibre density of anterior (**k**) and posterior (**l**) aspects, 50% fibre density of anterior (**m**) and posterior (**n**) aspects, and 100% fibre density of anterior (**o**) and posterior (**p**) aspects. These structural representations of the heart visualise the helical fibre orientation of cardiomyocytes in both age groups. Scale bar = 20 mm.

**Fig. 4:**
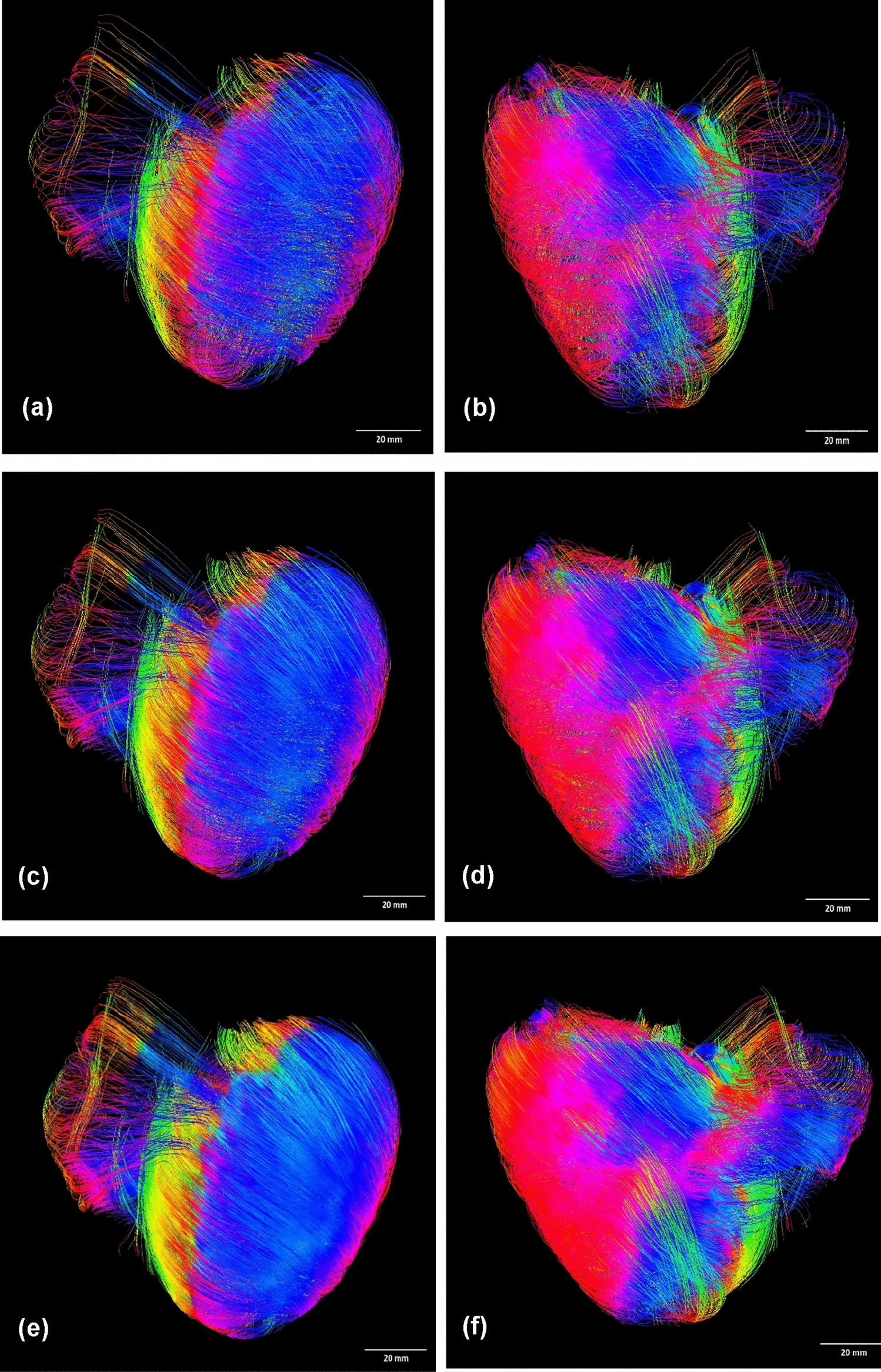

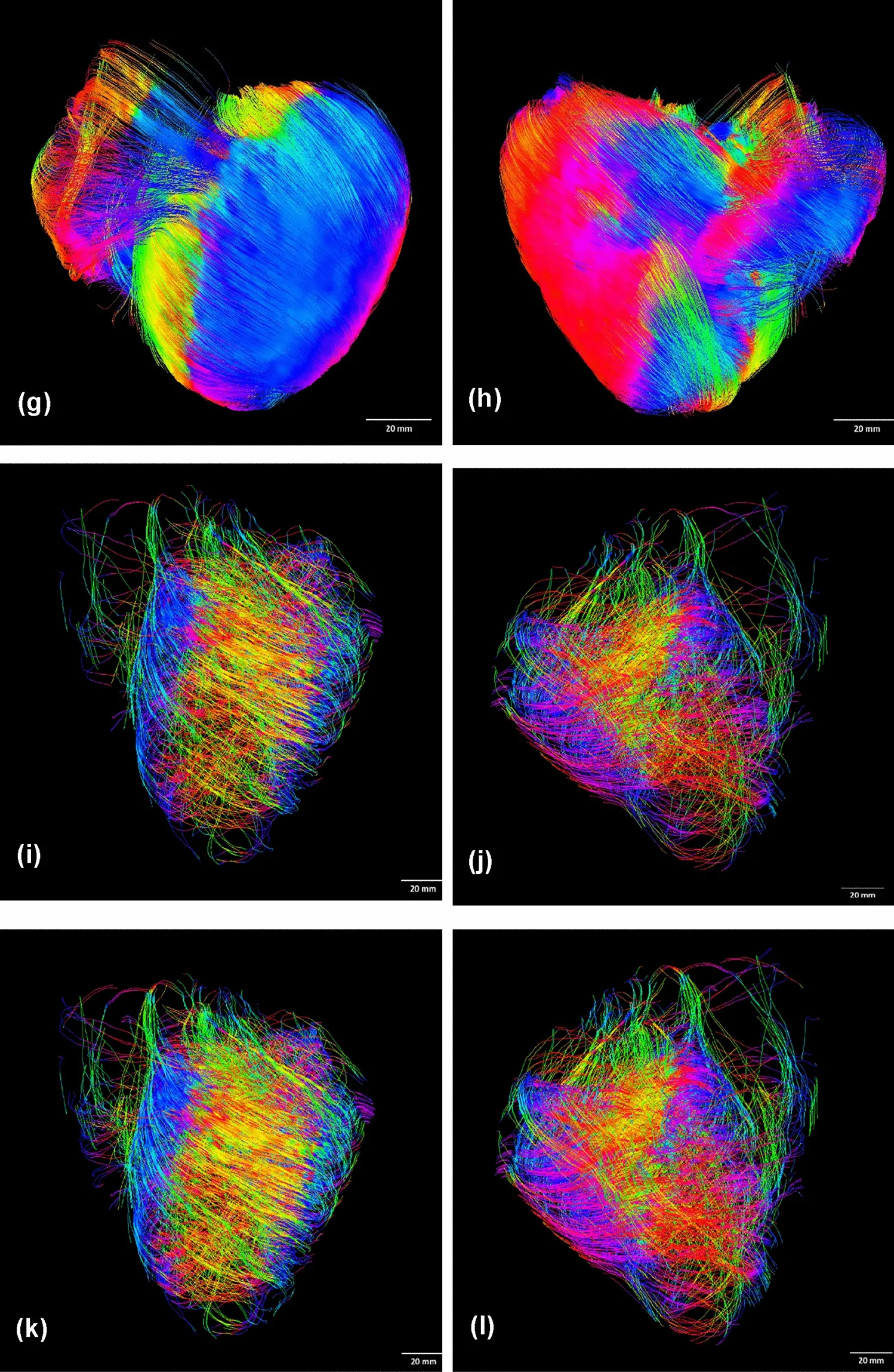

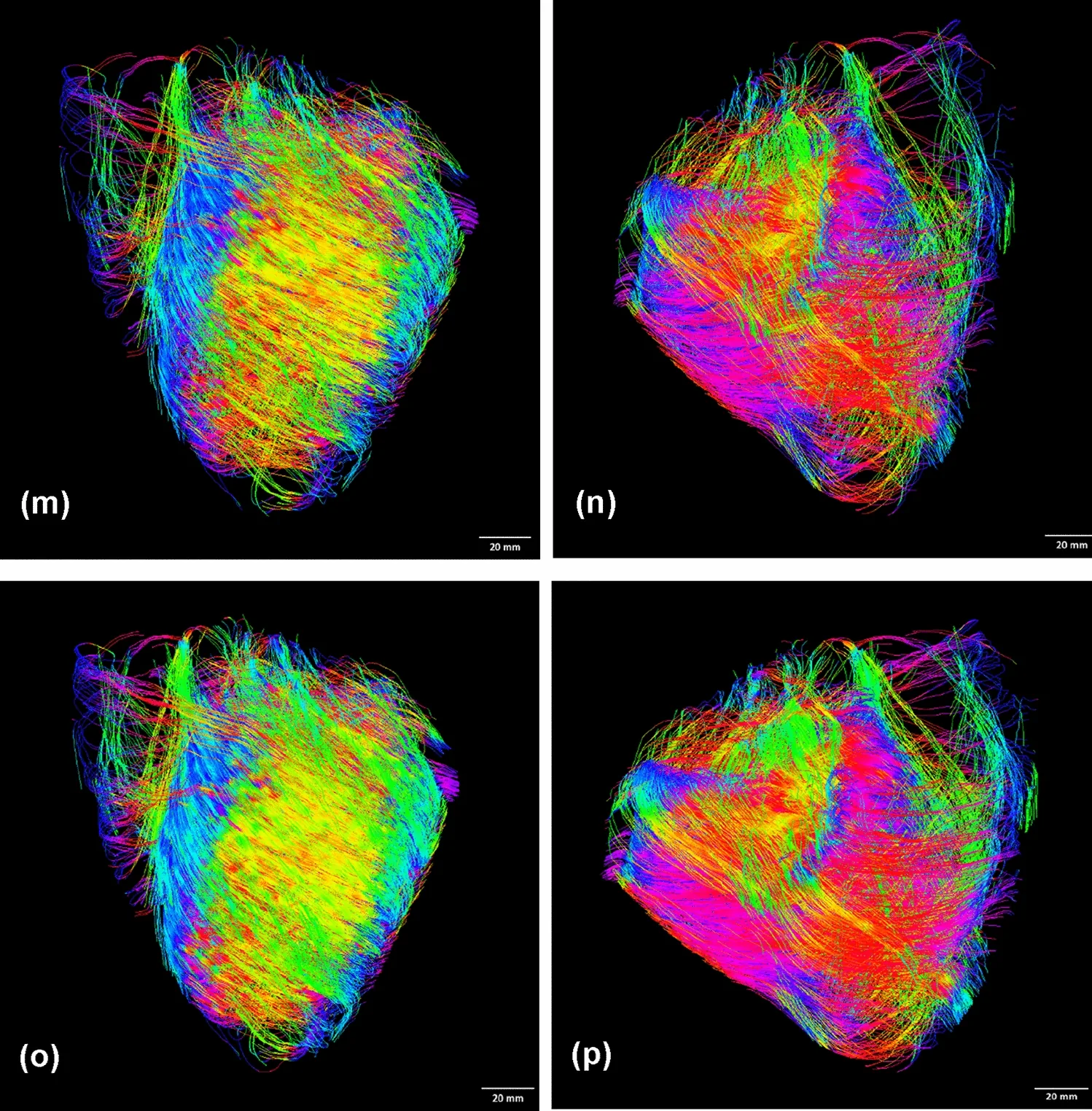
Post-processed DT-MRIs of a *7-month-old* porcine heart exhibiting 15% fibre density of anterior (**a**) and posterior (**b**) aspects, 25% fibre density of anterior (**c**) and posterior (**d**) aspects, 50% fibre density of anterior (**e**) and posterior (**f**) aspects, and 100% fibre density of anterior (**g**) and posterior (**h**) aspects. Post-processed DT-MRIs of a *3-year-old* porcine heart exhibiting 15% fibre density of anterior (**i**) and posterior (**j**) aspects, 25% fibre density of anterior (**k**) and posterior (**l**) aspects, 50% fibre density of anterior (**m**) and posterior (**n**) aspects, and 100% fibre density of anterior (**o**) and posterior (**p**) aspects. These structural representations of the heart visualise the helical fibre orientation of cardiomyocytes in both age groups. Scale bar = 20 mm

**Fig. 5:**
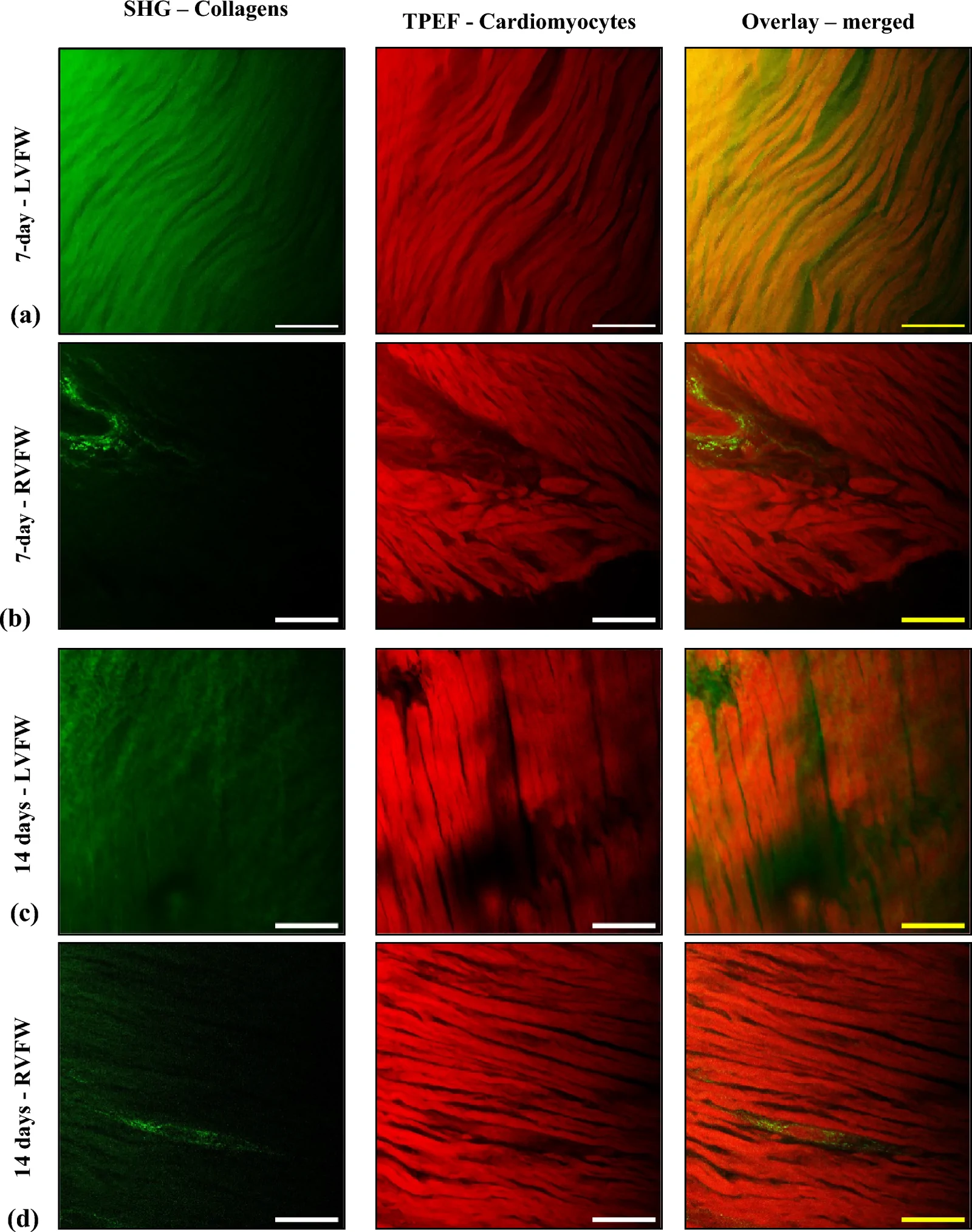

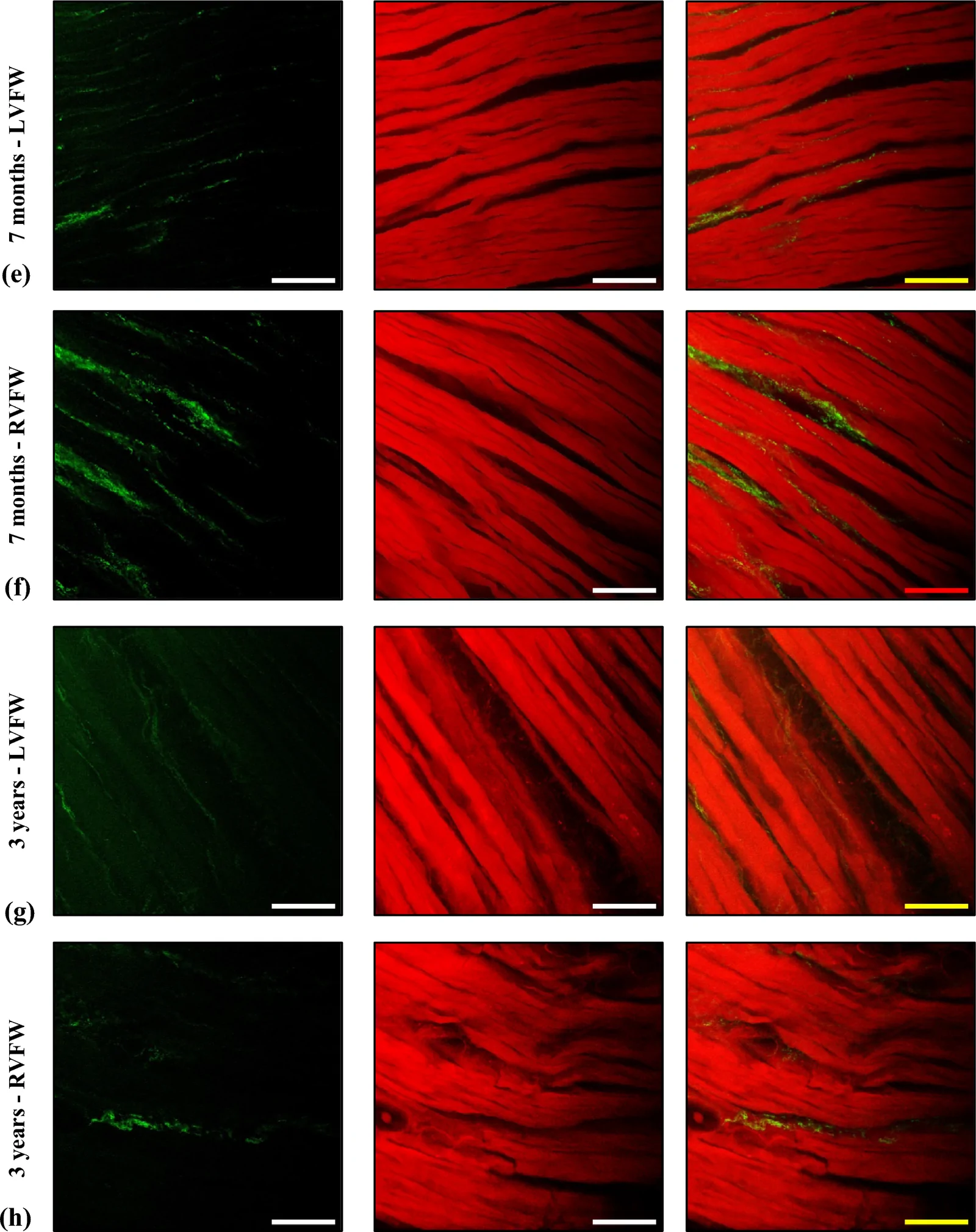
In-plane TPEF/SHG representative images of the porcine LVFW and RVFW. These representative images are taken from the equatorial region of the hearts. Four age groups are presented, namely: *7-day-old* LVFW (**a**) and RVFW (**b**); *14-day-old* LVFW (**c**) and RVFW (**d**); *7-month-old* LVFW (**e**) and RVFW (**f**); and *3-year-old* LVFW (**g**) and RVFW (**h**). The SHG channel (green) identifies the collagen fibril distribution, and the TPEF channel (red) identifies the cardiomyocyte. Both channels were merged to demonstrate the interaction between collagen and cardiomyocytes. A reference axis was defined as passing through the apex and base, with the edge of a 15 x15 mm square cutter kept parallel to this axis when dissecting tissue samples, taken through the ventricle walls. The defined reference axis (apex-to-base) is considered 0 °for all imaging Z-stacks. Z-stacks were obtained through the absolute thickness of the LVFW and RVFW (i.e., epicardium to endocardium). Scale bar = 30 μm. (For interpretation of the references to colour in this figure legend, the reader is referred to the web version of this article)

**Fig. 6:**
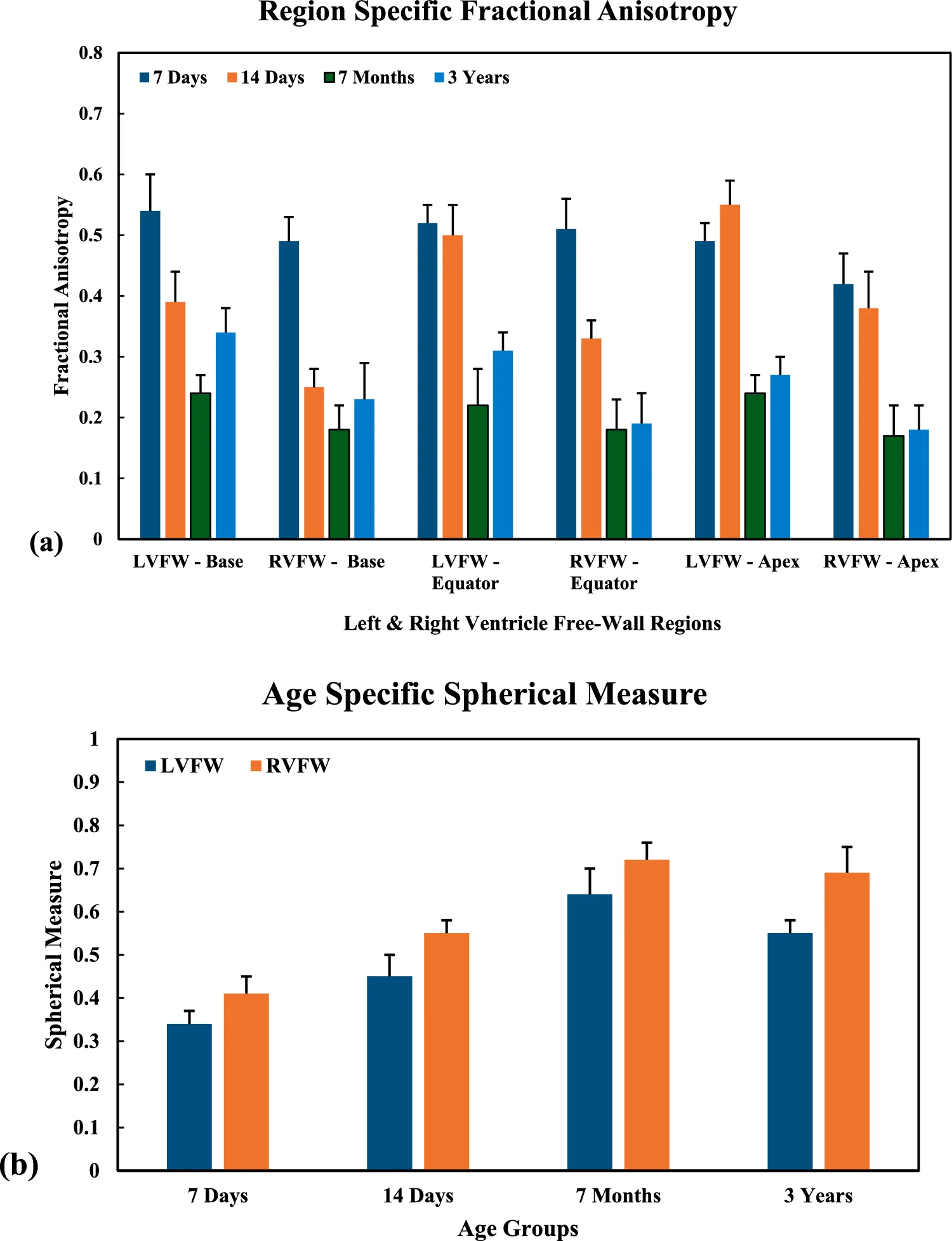
**a** Region-specific Fractional Anisotropy for the Left Ventricle ‘free-wall’ (LVFW) and Right Ventricle ‘free-wall’ (RVFW) across four age groups, namely 7-day-old, 14-day-old, 7-month-old, and 3-year-old, is demonstrated in the basal, equatorial, and apex regions of the ventricles. **b** Spherical Measure of the Left Ventricle ‘free-wall’ (LVFW) and Right Ventricle ‘free-wall’ (RVFW) is exhibited across four age groups, namely 7-day-old, 14-day-old, 7-month-old, and 3-year-old. All data is presented in mean values and corresponding standard deviation indicated by error bars

**Fig. 7:**
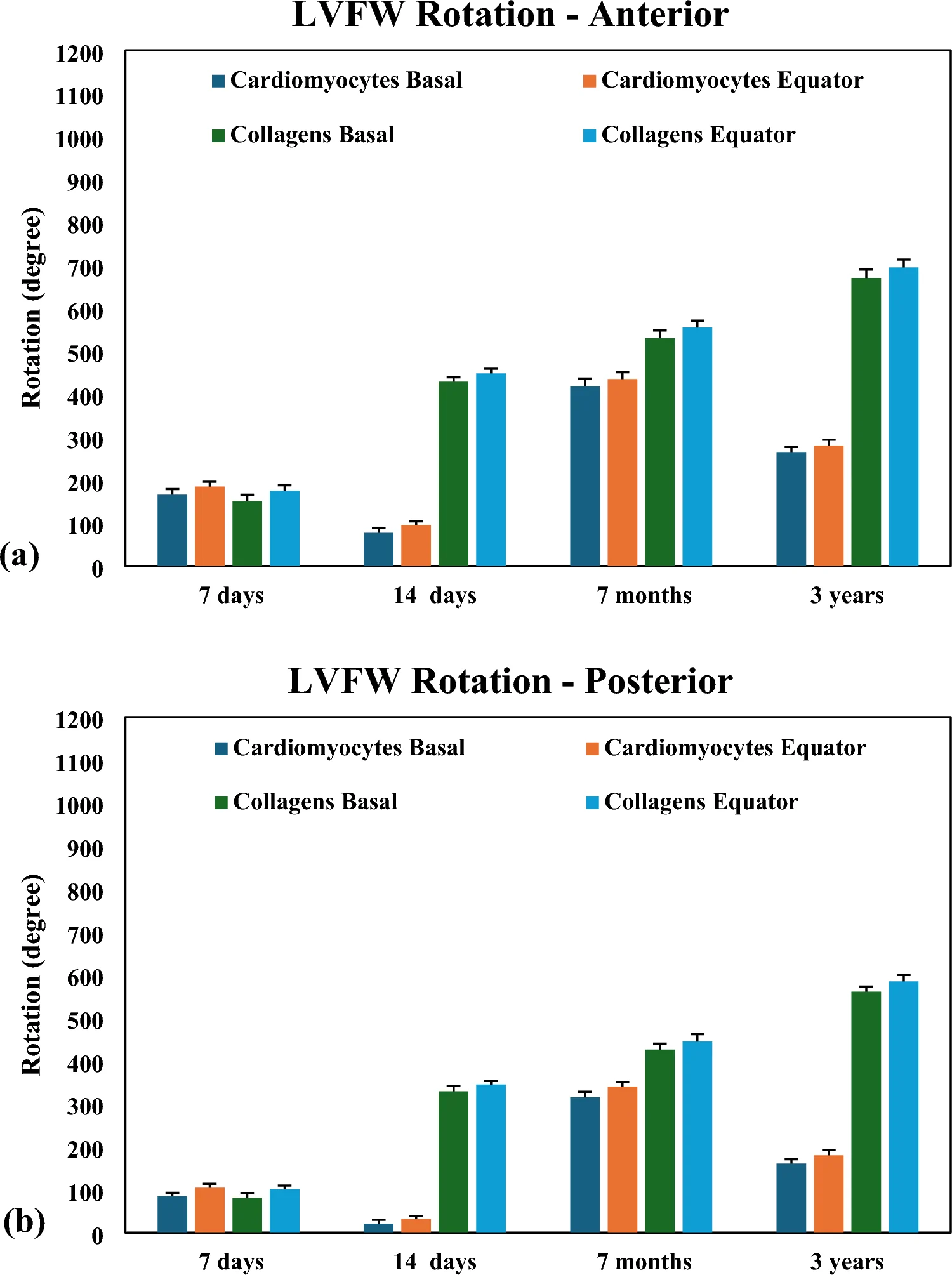

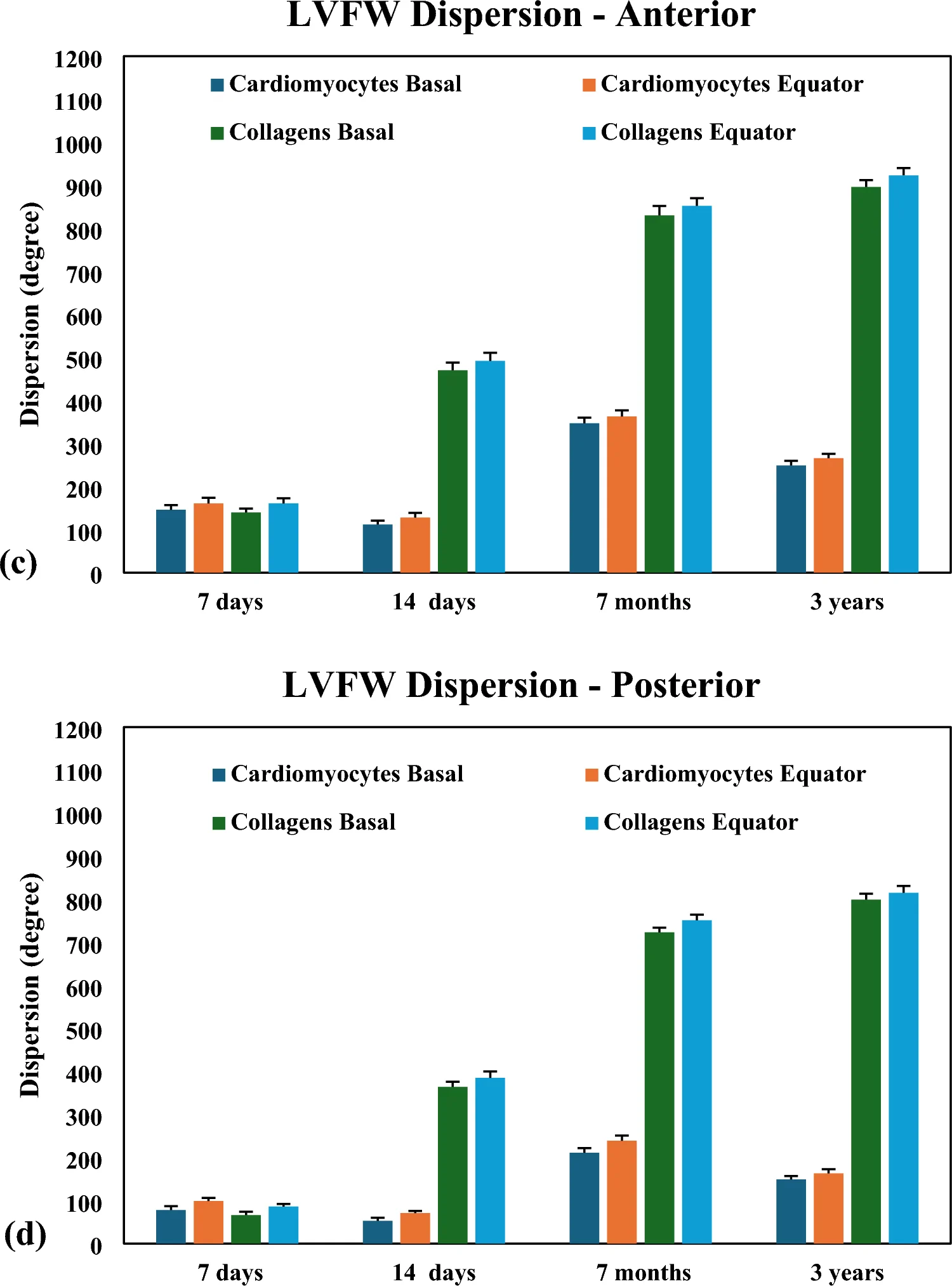
Left Ventricle ‘free wall’ (LVFW) cardiomyocytes and collagen fibrils rotation across four age groups, namely 7-day-old, 14-day-old, 7-month-old, and 3-year-old, is demonstrated in the basal and equatorial regions of the anterior (**a**) and posterior (**b**) aspects of the ventricle. Left Ventricle ‘free wall’ (LVFW) cardiomyocytes and collagen fibrils dispersion across four age groups, namely 7-day-old, 14-day-old, 7-month-old, and 3-year-old, is exhibited in the basal and equatorial regions of the anterior (**c**) and posterior (**d**) aspects of the ventricle. All data is presented in mean values and corresponding standard deviation indicated by error bars

**Fig. 8:**
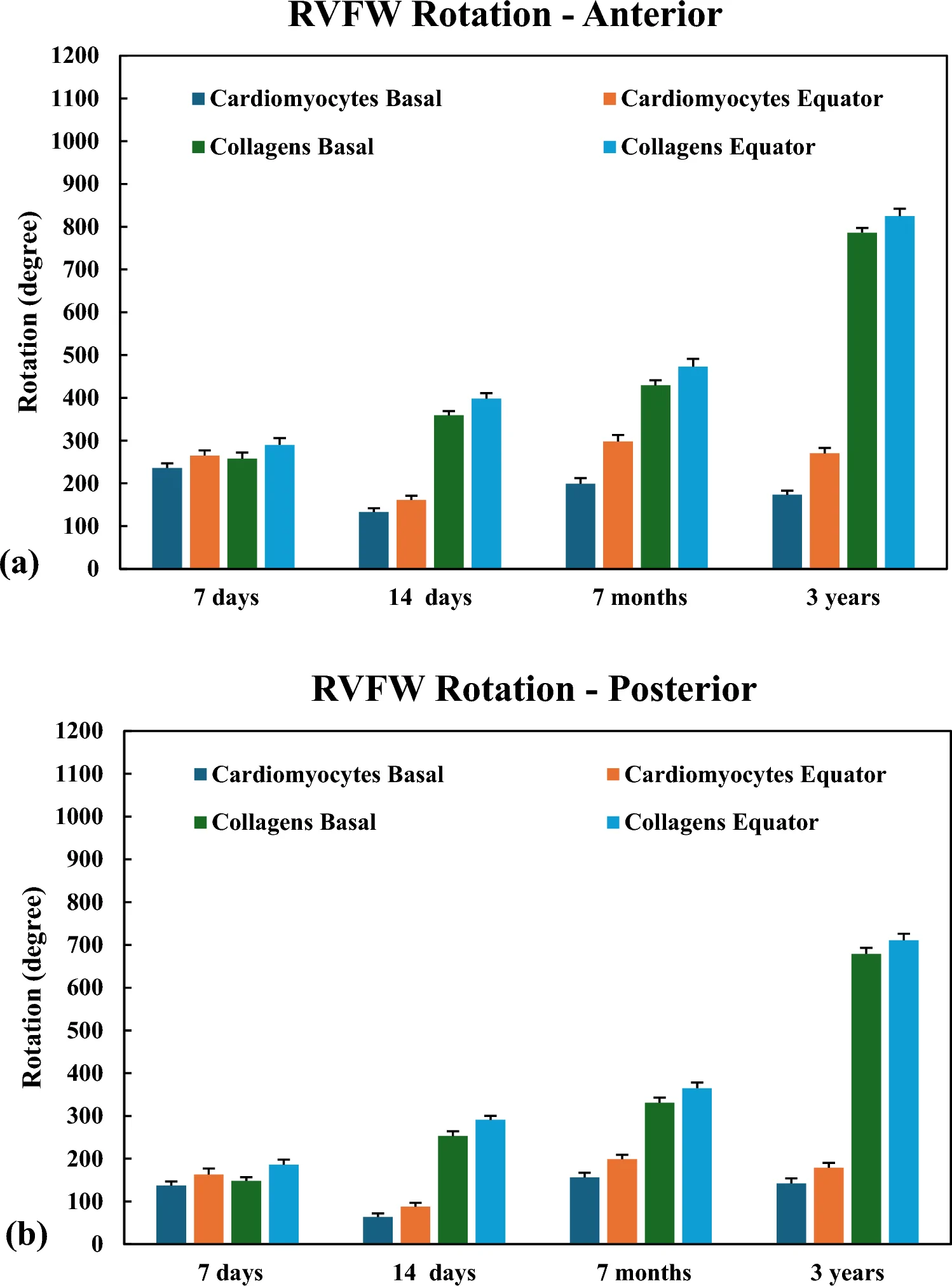

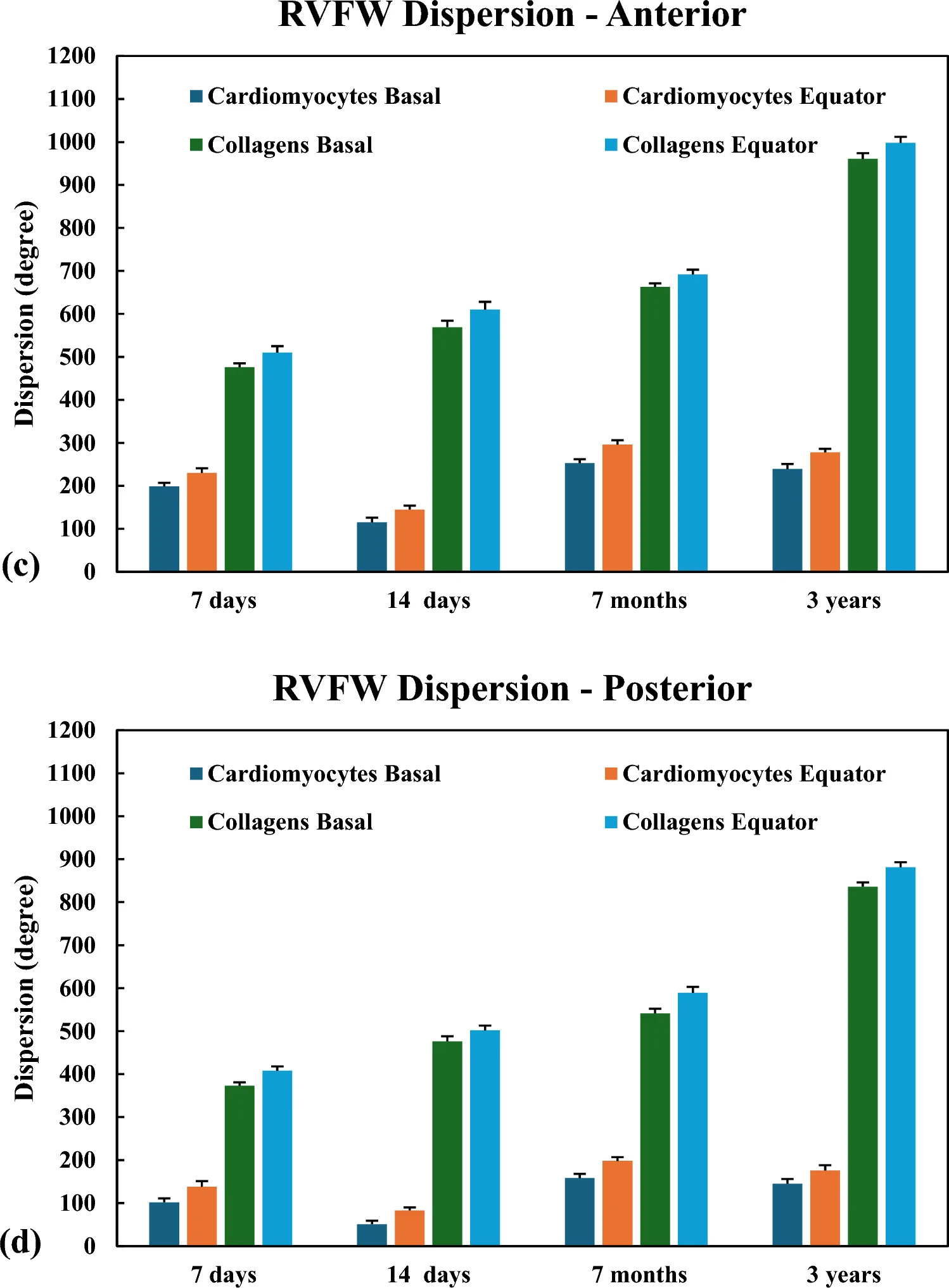
Right Ventricle ‘free wall’ (RVFW) cardiomyocytes and collagen fibrils rotation across four age groups, namely 7-day-old, 14-day-old, 7-month-old, and 3-year-old, is demonstrated in the basal and equatorial regions of the anterior (**a**) and posterior (**b**) aspects of the ventricle. Right Ventricle ‘free wall’ (RVFW) cardiomyocytes and collagen fibrils dispersion across four age groups, namely 7-day-old, 14-day-old, 7-month-old, and 3-year-old, is exhibited in the basal and equatorial regions of the anterior (**c**) and posterior (**d**) aspects of the ventricle. All data is presented in mean values and corresponding standard deviation indicated by error bars

Thirty-two female piglets and pigs (Landrace/Large White) were acquired at the following ages: 7 days (mass = 2.5 ± 0.4 kg), 14 days (mass = 5 ± 0.7 kg), 7 months (mass = 80 ± 10 kg), and 3 years (mass = 150 ± 20 kg) to give a large longitudinal range. Three hearts per age group (n = 3) were used for DT-MRI analysis, with a total of twelve hearts across four age groups. Five hearts per age group (n = 5) were used for TPEF/SHG analysis, with a total of twenty hearts across four age groups. Animals were maintained in accordance with institutional animal care policies and the UK Animals (Scientific Procedures) Act 1986 (ASPA). All procedures were authorised by the institutional Animal Welfare and Ethical Review Body (AWERB) under UIN UIN-22-08 (Histo-anatomical heart visualisation). At the predetermined experimental endpoint, piglets and pigs were humanely euthanised using a method permitted under Schedule 1 of ASPA for animals of this species and developmental stage. All euthanasia procedures were performed by trained and competent personnel in compliance with Home Office guidance. Immediately after confirmation of death, the hearts were collected using an aseptic technique (Figure 1). Samples were perfused with heparinised Phosphate buffer to ensure minimal post-mortem interval to maintain sample integrity (Fig. 2, 3, 4, 5, 6, 7, 8).

The left ventricle free-wall (LVFW) and right ventricle free-wall (RVFW) were then identified (Figure 2a), before defining the circumferential-radial-longitudinal (CRL) coordinate system. The longitudinal axis is defined vertically from the centre of the base to the apex. The circumferential axis is perpendicular to both the longitudinal and radial directions and is oriented in a counterclockwise direction around the short axis. The radial axis is directed perpendicular to the epicardial surface, pointing away from the ventricular epicardium (i.e., through thickness), as depicted in Figure 2a [55, 67].

### Methods

#### DT-MRI sample preparation

Three hearts (n = 3) per age group, with a total of twelve hearts across four age groups, were fixed in neutral-buffered formalin for 1-3 weeks. Following fixation, the hearts were suspended in 1% low-melting agarose to ensure an even agarose gel on all four planes. 1% low-melting agarose gel is used to fill heart chambers to prevent collapse during imaging. 1% low-melting agarose gel is a highly suitable and frequently used material for filling ventricles in MR imaging, as reported in the literature. This was then set for a minimum of 4 hours before placement into the MRI bore for image acquisition.

#### DT–MRI Image Acquisition and Analysis

DT-MRI was performed on a 3 T Prisma system (Siemens). A data volume of 96x96x36 mm was acquired with a voxel size of 1.8x1.8x1.8 mm. The GE diffusion tensor imaging protocol was used, with two b values (0, 1000) and 55 gradient directions [63]. Total acquisition time for each heart was ~ 3 hours.

3D Slicer software plug-in ‘SlicerDMRI’ was used to perform unscented Kalman filter tractography and scalar measurements. Diffusion-weighted images were used to calculate the diffusion tensor. The diffusion tensor D in each voxel was visualised as a diffusion ellipsoid. The eigenvectors were used to define the directions of the principal axes, and the ellipsoidal are proportional to the square root of the eigenvalues. The size and shape of the diffusion tensor were described by rotationally invariant eigenvalues λ_1_, λ_2_, and λ_3_. Diffusion tensor imaging was used to evaluate the trace and fractional anisotropy. Trace (D) and fractional anisotropy (FA) are scalar measures intrinsic to tissue and are independent of fibre orientation and diffusion sensitising gradient directions. Trace was used to calculate the size of the tensor, and fractional anisotropy characterised the shape (degree of ‘out of roundness’) of the diffusion ellipsoid, ranging from 0 (low FA) to 1 (high FA). The equation for Fractional Anisotropy (FA) calculations is defined in equation 1 [68, 69]. The tractography interactive seeding module was then used on the FA map to track the cardiomyocytes. Undesirable tracks were removed to obtain the required heart profile. In 3D Slicer, spherical measurements (SM) refer to shape analysis, which quantifies how closely a segmented 3D structure resembles a perfect sphere. It evaluates the roundness or sphericity of a 3D volume. A value of 1 indicates a perfect sphere, while values closer to 0 indicate a highly non-spherical shape. The equation for SM calculations is defined in equation 2. Both the FA and SM are scalar quantities. Finally, the regions of interest (ROIs) were selected (i.e., base, equator, and apex of LVFW and RVFW) to obtain regional FA and SM. This method is consistent with those described elsewhere [70].

1 $$\text{Fractional Anisotropy }(\mathrm{FA}) =\sqrt{\frac{3}{2}} \frac{\sqrt{{\lambda }_{1}-{\overline{ \lambda })}^{2}{+(\lambda }_{2}-{\overline{ \lambda })}^{2}{+(\lambda }_{3}-{\overline{ \lambda })}^{2}}}{\sqrt{{{\lambda }_{1}}^{2}+{{ \lambda }_{2}}^{2}+{{ \lambda }_{3}}^{2}}}$$

where, ${\lambda }_{1}$, ${\lambda }_{2}$, ${\lambda }_{3}$: The three eigenvalues of the diffusion tensor represent the magnitude of diffusion along the principal axes.

$\overline{\lambda }$ : The mean diffusivity, calculated as the average of the eigenvalues.

$$\overline{\lambda }=\frac{{\lambda }_{1}+ {\lambda }_{2}+ {\lambda }_{3}}{3};$$

2 $$\text{spherical measurement }=\frac{Surface area of a sphere with same Feret diameter}{Actual surface area of the segment}$$

where, Feret Diameter (D_F_)**:** The maximum distance between any two points on the object’s boundary.

Surface Area (A)**: **The total surface area of the 3D model, calculated from a mesh representation.

#### TPEF/SHG sample preparation

Five hearts (n = 5) per age group, with a total of twenty hearts across four age groups, were used for LVFW and RVFW specimens, which were dissected from the basal and equatorial region of the anterior and posterior aspects, as shown in Fig. 2b, c. A reference plane was defined as passing through the circumferential and longitudinal axes, with the edge of a 15 x15 mm square cutter kept parallel to these axes when dissecting tissue samples taken through the ventricle’s walls (Figure 2b). The circumferential axis is considered 0° for all imaging Z-stacks. Each dissected myocardial tissue sample was labelled with its heart number, region and age as a control parameter for handling multiple samples from the same heart, before being stored in 1000 mL cardioplegic solution with dissolved 20 mM 2, 3-butanedione monoxime (BDM), to inhibit cross-bridge activity and prevent any muscular contraction and stored at 4 °C [55]. The myocardial tissue walls were then sectioned into multiple thin specimens of 200 μm, via a sledge microtome (HM440E + K400 (Mircrom)), to image through the entire ventricle thickness (i.e., epicardium to endocardium).

#### Optical clearing of heart tissues for TPEF/SHG imaging

Myocardial samples were fixed with 4% paraformaldehyde (PFA) for 24 hours to achieve sufficient fixation. The myocardial samples were then rinsed with phosphate-buffered saline to wash off the PFA, then dehydrated using a graded ethanol series with each step lasting 4 hours, according to the following protocol: 50%, 70%, twice at 95% and twice at 100% [71]. A solution of 1:2 benzyl alcohol: benzyl benzoate (BABB) was used for optical clearing. Each sample was initially submerged in a solution of 1:1 ethanol: BABB for 24 hours before submerging into a 100% BABB solution, where it remained clear for at least 24 hours before imaging [71]. All steps involved in the tissue preparation, fixation and optical clearing were performed at room temperature.

#### TPEF/SHG Image Acquisition and Analysis

Tissue samples were mounted onto coverslips (VWR Scientific Inc.) and immersed in wintergreen oil (mountant). TPEF/SHG images were acquired by non-linear microscopy (NLM), using a laser scanning microscope (LSM880 NLO, Carl Zeiss, Ltd., Cambridge, UK) equipped with an ultrafast-pulsed, near-infrared TiS laser illumination system (Chameleon Vision II, Coherent Lasers, Cambridge, UK). Laser excitation at 900 nm and an approximate 140 fs pulse width were used for all NLM imaging, which was passed to the specimen and separated from returning emissions by a 690 nm short-pass primary dichroic reflector [72].

All NLM imaging was performed using this technique via an objective lens (Plan-Apochromat 63x/1.40 Oil Ph3, Zeiss). Backwards- propagating TPEF and SHG light from the specimen was collected by the objective and detected in the reflected light (epi–) pathway of the microscope, using the internal spectrometer to select the desired wavelengths. SHG (at half the excitation wavelength) was detected at 450 ± 10 nm, and TPEF at all wavelengths longer than 470 nm [72].

Two-channel (TPEF and SHG) 8-bit images were acquired simultaneously at serial focal positions to build a 3D stack of optical sections, collected at a rate of 1.52 μs. This comprised in-plane (*x, y)* stacks, with a field of view of 1024×1024 μm. Each line of every 2D optical section was scanned 16 times, and the average signal was recorded. The laser power during the acquisition of deeper images was automatically increased following a pre-set pattern to compensate for light scattering, reducing the illumination.

Tissue microstructure is typically characterised by quantifying preferred fibre orientation, fibre dispersion, fibre angles, and fibre rotation from TPEF/SHG imaging. Fiji/Image J (NIH, USA) software was used to perform quantitative analysis on TPEF/SHG image Z-stacks. In-plane (x, y) images were pre-processed in three dimensions using selected computational filters (unsharp mask, Gaussian blur 3D and Kuwahara) before these ‘stacks’ were analysed using the Fourier components analysis method [73]. This approach enabled the quantification of collagen and cardiomyocyte distributions relative to the stack depth. Using the ImageJ plug-in ‘Directionality’ (https://imagej.net/Directionality), data from all images within the stacks were used to generate a histogram. The peak was then fitted to a Gaussian function, enabling the identification of the preferred fibre orientation direction, a method consistent with other studies [74, 75]. The output comprised the preferred fibre orientation direction (°), defined by the centre of the Gaussian distribution, and the angular dispersion (°), defined as the standard deviation (std) of the Gaussian distribution.

Preferred fibre orientation refers to the aligned arrangement of fibres within the Z stacks of myocardial tissue, where most of the fibres are oriented within a specific reference plane (Figure 2d). The Circumferential – Longitudinal reference plane is used in our study as described in the Materials section and explained in Figure 2d. Fibre angles are calculated by analysing the changes in preferred fibre orientation direction within the Z stacks from epicardium to endocardium with respect to a Circumferential – Longitudinal reference plane, as demonstrated in the schematic diagram of Figure 2d. The circumferential axis is considered 0° for all imaging Z-stacks. A representative 7 months – LVFW preferred fibre orientation and angular dispersion from epicardium to endocardium is presented in the Supplementary Figure A.1a–d. Subsequently, the average fibre angles and dispersion angles from epicardium to endocardium are also demonstrated in the Supplementary Figure A.1e–f.

The cumulative absolute values (non-negative magnitude) of fibre ‘Rotation’ are then calculated from the changes in fibre angles within the Z stacks from epicardium to endocardium with respect to the circumferential axis, within the Circumferential – Longitudinal reference plane (Figure 2d). The fibre rotation is the extent/magnitude of fibre angles at which they change within each section of the Z stack from epicardium to endocardium as absolute values (positive numbers). Hence, the greater the Fibre angle variations, the higher the value of rotation would be within that stack from epicardium to endocardium.

### Statistical Analysis

The multiple regions of the hearts were averaged to one value per region for each heart before calculating the average values and standard deviation (SD) across the sample size (n). All values were reported as mean ± SD. Statistical analysis was performed using IBM SPSS Statistics (Version 30.0). Regional differences for average myocardial Fractional anisotropy (FA), Spherical measurements (SM), fibre angles, rotations, and dispersion were analysed using a linear mixed-effects model to account for repeated measurements within each heart, and to consider within-heart dependence and between-heart variability.

In the model, *Region* (Basal vs Equatorial vs Apex), *Ventricle* (Left vs Right), and *Wall* (Anterior vs Posterior) were considered as within-heart factors, whilst *age-group* (7 days, 14 days, 7 months, 3 years) was included as a fixed between-heart factor.

*Heart ID* (i.e., number) was considered as a random intercept to account for inter-heart variability.

Interaction effects between spatial factors and age (e.g., region x age and ventricle x age) were tested to assess developmental changes in myocardial structure. Post hoc pairwise comparisons were performed using Bonferroni correction. Statistical significance was set at *p* < 0.05.

## Results

### DT-MRI Analysis

DT-MRI enabled the visualisation of the gross anatomy for the anterior and posterior aspects across four age groups of the hearts, with quantification of fibre tractography, FA, and SM.

#### Fibre tractography, Fractional anisotropy (FA), and Spherical measurements (SM)

##### LVFW and RVFW

Fibre tractography was performed on DT-MRI images to obtain the three-dimensional helical structure of cardiomyocytes within the heart walls across all age groups (Figs. 3 and 4a–p). In younger hearts (7-day and 14-day-old), cardiomyocytes were aligned near diagonally (relative to the defined vertical axis of the heart, passing through the apex and base) in the anterior wall (Fig. 3a, c, e, g, i, k, m, and o). Cardiomyocytes are aligned near-horizontally in the posterior wall (Fig. 3b, d, f, h, j, l, n, and p). Cardiac myocyte fibres were aligned near diagonally in both the anterior and posterior aspects of the adult hearts (7 months and 3 years), as demonstrated in Fig. 4a–p. In younger hearts, cardiomyocyte alignment was predominantly prominent in parallel towards the lower basal and equatorial regions of the anterior and posterior aspects; alignment appeared weaker in the apex (Fig. 3a–p)***.*** In contrast, cardiomyocyte alignment was equally prominent in the basal, equatorial, and apex regions of the anterior and posterior aspects of the adult hearts (Fig. 4a–p).

FA varied within the LVFW, being greatest in the basal region, followed by the equator and apex across all four age groups of the heart (Table 1 and Figure 6a). This trend is consistent with RVFW (Table 1 and Figure 6a). LVFW possessed greater FA than RVFW across all four age groups of the heart (Table 1 and Figure 6a). The FA variations were also noted across four age groups, being greatest in 7 days and smallest in 7 months for both ventricles (Table 1 and Figure 6a). A statistical significance was found between the FA of three age groups, including 7 days, 7 months, and 3 years, for both ventricles (Table 1). There was no significant difference in the FA of LVFW and RVFW across the four age groups (p > 0.05) (Table 1, Figure 5).

**Table 1:** The regional Fractional Anisotropy (FA) of the anterior Left ventricle free-wall (LVFW) and Right ventricle free-wall (RVFW) of the porcine hearts at each age group, namely, 7 days, 14 days, 7 months and 3 years old, respectively

Regional Fractional Anisotropy (FA)	7 days		14 days		7 months	3 years
LVFW						
Basal		0.55 ± 0.04^a^		0.50 ± 0.05	0.24 ± 0.03^a^	0.34 ± 0.04
Equatorial		0.52 ± 0.05^a^		0.47 ± 0.04	0.22 ± 0.03^a^	0.31 ± 0.02
Apex		0.49 ± 0.03^a^		0.45 ± 0.05	0.20 ± 0.04^a^	0.27 ± 0.03
RVFW						
Basal	0.51 ± 0.05^a^			0.38 ± 0.02	0.18 ± 0.02^a^	0.23 ± 0.03
Equatorial	0.49 ± 0.04^a^			0.33 ± 0.05	0.16 ± 0.03^a^	0.19 ± 0.02
Apex	0.42 ± 0.03^a^			0.25 ± 0.04	0.14 ± 0.02^a^	0.17 ± 0.04

Results are expressed as mean ± standard deviation (n = 3)

^a^Linear Mixed-Effects Model revealed statistical significance across all age groups within the LVFW and RVFW, p < 0.05.

^b^Linear Mixed-Effects Model revealed statistical significance between the equivalent age groups across the LVFW and RVFW, p < 0.05.

Spherical measurements (SM) varied within the LVFW, being greatest in the basal region, followed by the equator and apex across all four age groups of the heart (Table 2 and Figure 6b). This trend is consistent with RVFW (Table 2 and Figure 6b). RVFW possessed greater SM than LVFW across all four age groups of the heart (Table 2 and Figure 6b). The SM variations were also noted across the four age groups, being greatest in 7 months and smallest in 7 days for both ventricles (Table 2 and Figure 6b). There was a significant difference between the SM across three age groups, including 7 days, 7 months, and 3 years, for both ventricles (Table 2). Statistical significance was not found between the SM of LVFW and RVFW across four age groups (p > 0.05) (Table 2).

**Table 2:** The regional Spherical Measure (SM) of the anterior Left ventricle free-wall (LVFW) and Right ventricle free-wall (RVFW) of the porcine hearts at each age group, namely, 7 days, 14 days, 7 months and 3 years old respectively

Regional Spherical Measure (SM)	7 days		14 days		7 months	3 years
LVFW						
Basal		0.38 ± 0.05^a^		0.45 ± 0.03	0.65 ± 0.04^a^	0.60 ± 0.02
Equatorial		0.34 ± 0.04^a^		0.36 ± 0.05	0.63 ± 0.02^a^	0.55 ± 0.03
Apex		0.27 ± 0.05^a^		0.29 ± 0.04	0.61 ± 0.03^a^	0.51 ± 0.02
RVFW						
Basal	0.46 ± 0.02^a^			0.62 ± 0.03	0.75 ± 0.02^a^	0.71 ± 0.03
Equatorial	0.40 ± 0.05^a^			0.53 ± 0.04	0.73 ± 0.02^a^	0.70 ± 0.03
Apex	0.36 ± 0.04^a^			0.51 ± 0.02	0.72 ± 0.04^a^	0.65 ± 0.02

Results are expressed as mean ± standard deviation (n = 3)

^a^Linear Mixed-Effects Model revealed statistical significance across all age groups within the LVFW and RVFW, p < 0.05

^b^Linear Mixed-Effects Model revealed statistical significance between the equivalent age groups across the LVFW and RVFW, p < 0.05

### TPEF/SHG Analyses

TPEF/SHG channels were used to identify the cardiomyocytes and collagen fibrils within the anterior and posterior aspects of the LVFW and RVFW across four age groups. The SHG channel, visualised in green, identified the collagen fibril distribution, whilst the TPEF channel in red highlighted cardiomyocyte distribution (Figure 5). Merging the two channels evaluated the in-plane cardiomyocyte and collagen region-specific (i.e., basal and equatorial) rotation (Figs. 7a, b, and 8a, b) and angular dispersion (Figs. 7a, b, and 8a, b) through the anterior and posterior aspects of the LVFW and RVFW across four age groups.

#### Cardiomyocytes and collagen fibril rotation and angles

In the LVFW, cardiomyocytes and collagen fibrils exhibited different rotations between the basal and equatorial regions across four age groups in the anterior and posterior aspects (Tables 3 and 4 and Fig. 7a, b). LVFW possessed greater cardiomyocyte and collagen fibril rotations in the equator than in the basal region across four age groups in the anterior and posterior aspects (Tables 3 and 4 and Fig. 7a, b). The anterior LVFW exhibited greater cardiomyocyte and collagen fibril rotation than the posterior between the basal and equator regions and across all age groups (Tables 3 and 4 and Fig. 7a, b). The LVFW cardiomyocyte rotation variations were also noted in the anterior and posterior walls across four age groups, with the greatest at 7 months and the smallest at 14 days. Initially, it decreases from 7 to 14 days, followed by an increase between 14 days and 7 months and finally decreases between 7 months and 3 years (Tables 3 and 4 and Fig. 7a, b)**.** In contrast to LVFW cardiomyocytes, the greatest and smallest LVFW collagen rotation was found at the age groups of 3 years and 7-day-old, respectively (Tables 3 and 4 and , b). It increased prominently from 1 day to 3 years old hearts (Tables 3 and 4 and Fig. 7a, b). This trend is consistent in the anterior and posterior LVFW.

**Table 3:** In-plane (x-y) cardiomyocytes and collagen fibril rotation and dispersion through the *LVFW - Anterior* (i.e. epicardium to endocardium) of the porcine heart at each age group, namely, 7 days, 14 days, 7 months and 3 years old respectively

LVFW - Anterior	7 days		14 days		7 months	3 years
Cardiomyocytes Basal						
Rotation (°)		166 ± 13^a, b^		77 ± 11^a, b^	418 ± 18^a, b^	265 ± 12^a, b^
Dispersion (°)		146 ± 11^a, b^		112 ± 9	347 ± 13^a, b^	249 ± 11^a^
Cardiomyocytes Equator						
Rotation (°)	185 ± 11^a, b^		95 ± 9^a, b^		435 ± 16^a, b^	280 ± 14^a^
Dispersion (°)	161 ± 13^a, b^		128 ± 11^a, b^		363 ± 14^a, b^	278 ± 10^a, b^
Collagens Basal						
Rotation (°)	151 ± 15^a, b^		429 ± 10^a, b^		530 ± 18^a, b^	670 ± 20^a, b^
Dispersion (°)	140 ± 9^a, b^		470 ± 18^a, b^		830 ± 22^a, b^	896 ± 16^a, b^
Collagens Equator						
Rotation (°)	175 ± 13^a, b^		448 ± 11^a, b^		555 ± 16^a, b^	695 ± 18^a, b^
Dispersion (°)	161 ± 12^a, b^		492 ± 19^a, b^		852 ± 18^a, b^	923 ± 17^b^

Results are expressed as mean ± standard deviation (n = 5)

^a^Linear Mixed-Effects Model revealed statistical significance across all age groups within the LVFW and RVFW, p < 0.05

^b^Linear Mixed-Effects Model revealed statistical significance between the equivalent age groups across the LVFW and RVFW, p < 0.05

**Table 4:** In-plane (x-y) cardiomyocytes and collagen fibril rotation and dispersion through the *LVFW - Posterior* (i.e. epicardium to endocardium) of the porcine heart at each age group, namely, 7 days, 14 days, 7 months and 3 years old respectively

LVFW - Posterior	7 days		14 days	7 months	3 years
Cardiomyocytes Basal					
Rotation (°)		85 ± 8^a, b^	21 ± 9^b^	315 ± 13^a, b^	161 ± 10^a, b^
Dispersion (°)		78 ± 9	51 ± 7	211 ± 11^a, b^	149 ± 8^a^
Cardiomyocytes Equator					
Rotation (°)	105 ± 9^a, b^		32 ± 9^a, b^	340 ± 11^a, b^	180 ± 13
Dispersion (°)	99 ± 7^a, b^		71 ± 5^b^	239 ± 12^a, b^	176 ± 10^a^
Collagens Basal					
Rotation (°)	81 ± 11^a, b^		329 ± 13^a, b^	426 ± 14^a, b^	561 ± 12^a, b^
Dispersion (°)	66 ± 8^a, b^		364 ± 12^a, b^	723 ± 11^a, b^	799 ± 14^a, b^
Collagens Equator					
Rotation (°)	101 ± 9^a, b^		345 ± 8^a, b^	445 ± 17^a, b^	585 ± 15^a, b^
Dispersion (°)	86 ± 6^a, b^		385 ± 15^a, b^	751 ± 13^a, b^	815 ± 16^b^

Results are expressed as mean ± standard deviation (n = 5)

^a^Linear Mixed-Effects Model revealed statistical significance across all age groups within the LVFW and RVFW, p < 0.05

^b^Linear Mixed-Effects Model revealed statistical significance between the equivalent age groups across the LVFW and RVFW, p < 0.05

In the RVFW, cardiomyocytes and collagen fibrils demonstrated different rotations between the basal and equatorial regions across four age groups in the anterior and posterior aspects (Tables 5 and 6 and Fig. 8a, b). RVFW exhibited greater cardiomyocyte and collagen fibril rotations in the equator than in the basal region across four age groups in the anterior and posterior aspects (Tables 5 and 6 and Fig. 8a, b). The anterior RVFW possessed greater cardiomyocyte and collagen fibril rotation than the posterior between the basal and equator regions and across four age groups (Tables 5 and 6 and Fig. 8a, b). The variations in RVFW cardiomyocyte rotation were also noted in the anterior and posterior walls across four age groups, with the greatest at 7 months and the smallest at 14 days. It initially decreases from 7 to 14 days, followed by an increase between 14 days and 7 months and finally decreases between 7 months and 3 years (Tables 5 and 6 and Fig. 8a, b)**.** Contrary to RVFW cardiomyocytes, the greatest and smallest RVFW collagen rotation was found at the age groups of 3 years and 7-day-old, respectively (Tables 5 and 6 and Fig. 8a, b). It increased notably from 1 day to 3 years old hearts (Tables 5 and 6 and Fig. 8a, b). This trend is consistent in the anterior and posterior RVFW.

**Table 5:** In-plane (x-y) cardiomyocytes and collagen fibril rotation and dispersion through the *RVFW – Anterior* (i.e. epicardium to endocardium) of the porcine heart at each age group, namely, 7 days, 14 days, 7 months and 3 years old respectively

RVFW - Anterior	7 days		14 days		7 months	3 years
Cardiomyocytes Basal						
Rotation (°)		236 ± 11^a, b^		133 ± 9^a, b^	199 ± 13^b^	173 ± 10^b^
Dispersion (°)		199 ± 8^a, b^	115 ± 11		253 ± 9^a, b^	239 ± 12
Cardiomyocytes Equator						
Rotation (°)	265 ± 12^a, b^		161 ± 10^a, b^		298 ± 15^b^	270 ± 13
Dispersion (°)	230 ± 11^a, b^		145 ± 9^a, b^		296 ± 10^a, b^	266 ± 8^b^
Collagens Basal						
Rotation (°)	258 ± 14^a, b^		359 ± 10^a, b^		429 ± 12^a, b^	786 ± 11^a, b^
Dispersion (°)	476 ± 9^a, b^		569 ± 15^a, b^		663 ± 8^a, b^	961 ± 13^a, b^
Collagens Equator						
Rotation (°)	290 ± 16^a, b^		398 ± 13^a, b^		473 ± 18^a, b^	825 ± 17^a, b^
Dispersion (°)	510 ± 15^a, b^		610 ± 18^a, b^		692 ± 11^b^	998 ± 14^a, b^

Results are expressed as mean ± standard deviation (n = 5)

^a^Linear Mixed-Effects Model revealed statistical significance across all age groups within the LVFW and RVFW, p < 0.05

^b^Linear Mixed-Effects Model revealed statistical significance between the equivalent age groups across the LVFW and RVFW, p < 0.05

**Table 6:** In-plane (x-y) cardiomyocytes and collagen fibril rotation and dispersion through the *RVFW – Posterior* (i.e. epicardium to endocardium) of the porcine heart at each age group, namely, 7 days, 14 days, 7 months and 3 years old respectively

RVFW - Posterior	7 days		14 days		7 months	3 years
Cardiomyocytes Basal						
Rotation (°)		137 ± 10^a, b^		64 ± 8^a, b^	156 ± 11^a, b^	142 ± 12^b^
Dispersion (°)		102 ± 9^a, b^	53 ± 8		158 ± 10^a, b^	145 ± 11
Cardiomyocytes Equator						
Rotation (°)	163 ± 14^a, b^		88 ± 9^a, b^		199 ± 10^a, b^	179 ± 11
Dispersion (°)	138 ± 13^a, b^		83 ± 7^a, b^		198 ± 9^a, b^	163 ± 12
Collagens Basal						
Rotation (°)	148 ± 9^a, b^		253 ± 11^a, b^		331 ± 12^a, b^	679 ± 14^a, b^
Dispersion (°)	373 ± 8^a, b^		476 ± 12^a, b^		541 ± 11^b^	836 ± 10^a, b^
Collagens Equator						
Rotation (°)	186 ± 12^a, b^		291 ± 9^a, b^		365 ± 13^b^	711 ± 15^a, b^
Dispersion (°)	408 ± 10^a, b^		502 ± 11^a, b^		589 ± 14^b^	881 ± 12^a, b^

Results are expressed as mean ± standard deviation (n = 5)

^a^Linear Mixed-Effects Model revealed statistical significance across all age groups within the LVFW and RVFW, p < 0.05

^b^Linear Mixed-Effects Model revealed statistical significance between the equivalent age groups across the LVFW and RVFW, p < 0.05

In the anterior and posterior walls, RVFW exhibited greater cardiomyocyte rotation in the younger hearts, whilst LVFW possessed greater cardiomyocyte rotation in the adult hearts, as reported in Tables 3, 4, 5 and 6. There was a significant difference in cardiomyocyte and collagen fibril rotation in the anterior and posterior aspects of both ventricles across four age groups (p < 0.05) (Tables 3, 4, 5 and 6). The anterior LVFW and RVFW possessed greater fibre angles compared to the posterior for cardiomyocytes and collagen fibrils in the epicardium, mid-wall, and endocardium regions across all age groups, as reported in Tables 7 and 8. The epicardium and endocardium regions of cardiomyocytes and collagen fibrils demonstrated greater average fibre angles than the mid-wall region across all age groups of LVFW and RVFW (Tables 7 and 8). LVFW fibre angles for cardiomyocytes and collagen fibril decreased from 7 days to 7 months and then increased until 3 years (Tables 7 and 8). Overall, LVFW exhibited greater cardiomyocyte and collagen fibre angles than RVFW in the anterior and posterior aspects across all age groups (Tables 7 and 8). There was no significant difference in cardiomyocyte and collagen average fibre angles in the anterior and posterior aspects of both ventricles across four age groups (p < 0.05) (Tables 7 and 8).

**Table 7:** Average cardiomyocytes and collagens fibre angles through the *LVFW - Anterior and Posterior* for equatorial regions in epicardium, mid-wall, and endocardium of the porcine heart at each age group, namely, 7 days, 14 days, 7 months and 3 years old respectively

LVFW	7 days	14 days	7 months	3 years
*LVFW - Anterior* Cardiomyocytes				
Epicardium Fibre Angle (°)	-59 ± 13	-46 ± 11	-32 ± 10	-54 ± 12
Mid-wall Fibre Angle (°)	30 ± 11	16 ± 9	17 ± 8	22 ± 10
Endocardium Fibre Angle (°)	34 ± 10	32 ± 11	30 ± 12	31 ± 13
Collagens				
Epicardium Fibre Angle (°)	-39 ± 11	-29 ± 9	-24 ± 10	-48 ± 14
Mid-wall Fibre Angle (°)	18 ± 9	11 ± 7	16 ± 9	27 ± 10
Endocardium Fibre Angle (°)	62 ± 16	28 ± 12	28 ± 11	29 ± 13
*LVFW - Posterior* Cardiomyocytes				
Epicardium Fibre Angle (°)	-50 ± 15	-39 ± 10	-28 ± 13	-42 ± 14
Mid-wall Fibre Angle (°)	21 ± 9	12 ± 7	14 ± 8	19 ± 9
Endocardium Fibre Angle (°)	28 ± 11	24 ± 12	22 ± 10	27 ± 13
Collagens				
Epicardium Fibre Angle (°)	-31 ± 13	-23 ± 11	-19 ± 8	-40 ± 17
Mid-wall Fibre Angle (°)	15 ± 7	10 ± 6	11 ± 7	19 ± 8
Endocardium Fibre Angle (°)	51 ± 12	21 ± 11	20 ± 10	22 ± 9

Results are expressed as mean ± standard deviation (n = 5)

^a^Linear Mixed-Effects Model revealed statistical significance across all age groups within the LVFW and RVFW, p < 0.05

^b^Linear Mixed-Effects Model revealed statistical significance between the equivalent age groups across the LVFW and RVFW, p < 0.05

**Table 8:** Average cardiomyocytes and collagens fibre angles through the *RVFW - Anterior and Posterior* for equatorial regions in epicardium, mid-wall, and endocardium of the porcine heart at each age group, namely, 7 days, 14 days, 7 months and 3 years old respectively

	7 days	14 days	7 months	3 years
*RVFW - Anterior* Cardiomyocytes				
Epicardium Fibre Angle (°)	-23 ± 13	-22 ± 11	-18 ± 10	-22 ± 9
Mid-wall Fibre Angle (°)	22 ± 11	21 ± 9	10 ± 6	9 ± 5
Endocardium Fibre Angle (°)	33 ± 11	33 ± 12	33 ± 10	45 ± 15
Collagens				
Epicardium Fibre Angle (°)	-22 ± 11	-21 ± 9	-10 ± 6	-18 ± 9
Mid-wall Fibre Angle (°)	23 ± 13	20 ± 11	9 ± 5	8 ± 4
Endocardium Fibre Angle (°)	15 ± 9	17 ± 10	33 ± 13	36 ± 16
*RVFW - Posterior* Cardiomyocytes				
Epicardium Fibre Angle (°)	-21 ± 11	-20 ± 10	-16 ± 8	-20 ± 12
Mid-wall Fibre Angle (°)	20 ± 9	19 ± 8	9 ± 5	8 ± 4
Endocardium Fibre Angle (°)	28 ± 13	26 ± 11	25 ± 16	34 ± 17
Collagens				
Epicardium Fibre Angle (°)	-19 ± 13	-17 ± 11	-8 ± 4	-14 ± 7
Mid-wall Fibre Angle (°)	18 ± 10	16 ± 8	8 ± 5	7 ± 3
Endocardium Fibre Angle (°)	13 ± 7	14 ± 6	23 ± 11	25 ± 12

Results are expressed as mean ± standard deviation (n = 5)

^a^Linear Mixed-Effects Model revealed statistical significance across all age groups within the LVFW and RVFW, p < 0.05

^b^Linear Mixed-Effects Model revealed statistical significance between the equivalent age groups across the LVFW and RVFW, p < 0.05

#### Cardiomyocytes and collagen fibril dispersion

In the LVFW, cardiomyocytes and collagen fibrils exhibited different dispersion between the basal and equatorial regions across four age groups in the anterior and posterior aspects (Tables 3 and 4 and Fig. 7c and 7d). LVFW possessed greater cardiomyocyte and collagen fibril dispersion in the equator than in the basal region across four age groups in the anterior and posterior aspects (Tables 3 and 4 and Fig. 7c, d). The anterior LVFW exhibited greater cardiomyocyte and collagen fibril dispersion than the posterior between the basal and equator regions and across four age groups (Tables 3 and 4 and Fig. 7c, d). The LVFW cardiomyocyte dispersion variations were also noted in the anterior and posterior walls across four age groups, with the greatest at 7 months and the smallest at 14 days. Firstly, it decreases from 7 to 14 days, followed by an increase between 14 days and 7 months and finally decreases between 7 months and 3 years (Tables 3 and 4 and Fig. 7c, d)**.** In contrast to LVFW cardiomyocytes, the greatest and smallest LVFW collagen dispersion was found at the age groups of 3 years and 7-day-old, respectively (Tables 3 and 4 and Fig. 7c, d). It increased prominently from 1 day to 3 years old hearts (Tables 3 and 4 and Fig. 7c, d). This trend is consistent in the anterior and posterior LVFW.

In the RVFW, cardiomyocytes and collagen fibrils demonstrated different dispersions between the basal and equatorial regions across four age groups in the anterior and posterior aspects (Tables 5 and 6 and Fig. 8c, d). RVFW exhibited greater cardiomyocyte and collagen fibril dispersion in the equator than in the basal region across four age groups in the anterior and posterior aspects (Tables 5 and 6 and Fig. 8c, d). The anterior RVFW possessed greater cardiomyocyte and collagen fibril dispersion than the posterior between the basal and equator regions and across four age groups (Tables 5 and 6 and Fig. 8c, d). The variations in RVFW cardiomyocyte dispersion were also noted in the anterior and posterior walls across four age groups, with the greatest at 7 months and the smallest at 14 days. It initially decreases from 7 to 14 days, followed by an increase between 14 days and 7 months and finally decreases between 7 months and 3 years (Tables 5 and 6 and Fig. 8c, d)**.** Contrary to RVFW cardiomyocytes, the greatest and smallest RVFW collagen dispersion was found at the age groups of 3 years and 7-day-old, respectively (Tables 5 and 6 and Fig. 8c, d). It increased notably from 1 day to 3 years old hearts (Tables 5 and 6 and Fig. 8c, d). This trend is consistent in the anterior and posterior RVFW.

RVFW possessed greater cardiomyocyte dispersion in the younger hearts, whilst LVFW exhibited greater cardiomyocyte dispersion in the adult hearts in the anterior and posterior aspects, as reported in Tables 3, 4, 5 and 6. There was a significant difference in cardiomyocyte and collagen fibril dispersion in the anterior and posterior aspects of both ventricles across four age groups (p < 0.05) (Tables 3, 4, 5 and 6).

## Discussion

DT-MRI and TPEF-SHG imaging modalities were used to describe the microstructural changes in the heart due to adaptive ageing across four age groups from neonatal to adulthood. These microstructural data report the region-specific (i.e., basal and equatorial) and age-dependent differences between the anterior and posterior aspects of the LVFW and RVFW. These microstructural differences provide valuable insight into heart ageing, due to its growth and development in response to a new oxygenation supply and increasing physiological demands.

Fibre tractography assessment revealed that cardiomyocyte alignment in the anterior LVFW was relatively strong, with fewer extracellular spaces relative to the posterior surface across four age groups (Figs. 3 and 4). The regional differences observed in the cardiomyocyte alignment and orientation, changing within the anterior and posterior LVFW, may contribute to the twisting and untwisting of the left ventricle during systole and diastole (Tables 3 and 4) [76]. This recoil mechanism is important because it determines the myocardial compliance and left ventricular suction, which regulates the diastolic function of the heart [77, 78].

The anterior wall has been reported to significantly contribute to the left ventricle’s twisting, compared to the posterior wall, during systolic heart function [79, 80]. Thus, the greater cardiomyocyte rotation in the anterior wall may provide structural support for generating force to eject blood (Tables 3 and 4 and Figs. 3 and 4). The relative increase in cardiomyocyte rotation in the anterior wall, with hypertrophy, was observed in the adult hearts, compared to the anterior and posterior aspects of younger hearts, in the basal and equatorial regions of both ventricles (Tables 3, 4, 5 and 6 and Figs. 3 and 4). This may be due to the combination of regional cell loss and changes in the extracellular matrix (ECM) composition, to facilitate the increased physiological demands, as previously reported in the literature [81, 82].

The cardiomyocyte architecture, including orientation and dispersion distribution, is known to change regionally within the ventricles, which may vary the regional (i.e. base, equator and apex) FA in the LVFW and RVFW, reported in this study (Tables 1, 3, 5 and Figure 6a) [83, 84]. Cardiomyocytes are organised in small functional units, known as sheets. The regional variations in cardiomyocyte rotation and dispersion within the sheets may also contribute to varying the regional FA (Table 1, 3, 5 and Figure 6a) [85, 86].

An inverse relationship between FA and fibre dispersion was noted in this study via DT-MRI and TPEF-SHG. This trend is consistent with the previously published literature [87, 88]. Younger hearts exhibited greater FA and smaller fibre dispersion than adults (Table 1, 3, 5 and Figure 6a). A two-fold increase in fibre dispersion and a reciprocal decrease in FA were observed during the developmental transition of young to adult hearts (Table 1, 3, 5 and Figure 6a). This may primarily be due to the maturation of the cardiac microarchitecture, which involves changing from a tightly packed, relatively simpler structure to a more complex, interwoven, and mature network in response to increased physiological demands on the heart (Tables 3, 4, 5, and 6) [89]. This could also, in part, be due to increased cardiomyocyte size and changed density during ECM development, as previously reported in the literature [90]. Hence, the transition from a simpler, compact and uniform fibre orientation in the young heart to a complex, mature 3D architecture in the adult, resulting in higher fibre dispersion and lower fractional anisotropy, as reported in this study (Table 1, 3, 5 and Figure 6a).

There was an increase in spherical measurement (SM) between the younger and adult hearts, indicating the geometrical changes in the ventricles with ageing (Table 2 and Figure 6b). These changes may be driven by increased systemic pressure [91], a shift from RVFW-dominant to LVFW-dominant circulation [92], maturation of fibres [93], contractile, and ECM, and torsional mechanics, as reported in the literature [94]. RVFW possessed a greater SM than the LVFW across four age groups (Table 2 and Figure 6b). The right ventricle is more spherical because it is a thin-walled, low-pressure, volume-handling chamber with relatively simpler fibre architecture (i.e., fibre angle, fibre rotation and dispersion) and septal dependence (Tables 3, 4, 5, 6, 7 and 8) [95, 96]. In contrast, the left ventricle is a thick-walled, high-pressure pump whose fibre structure and mechanics favour an ellipsoidal shape [95, 96].

The regional variations (i.e., basal and equatorial) in cardiomyocyte and collagen fibril rotation and dispersion were noted between the anterior and posterior aspects of both ventricles across four groups (Tables 3, 4, 5, and 6 and Figs. 7 and 8). This may be primarily due to a combination of regional mechanical forces (biomechanical stress/strain), developmental processes, and functional demands of the ventricles [97, 98]. Cardiomyocytes and collagen fibrils exhibited greater rotation and dispersion in the anterior adult hearts, compared to the anterior and posterior younger hearts (Tables 3, 4, 5, and 6 and Figs. 7 and 8). This could be due to age-related fibrotic remodelling, increased collagen cross-linking, and compensatory cardiomyocyte hypertrophy in response to cell loss and increased mechanical load, as previously reported [81]. These changes also cause increased myocardial stiffness in the adult ventricles [99]. The adult LVFW (i.e., 3 years old ∼265°) cardiomyocyte rotation reported here is similar to that observed in the previously published studies of human and porcine heart, reported in Table 3 [54, 55]. Additionally, the total cardiomyocyte rotation in the left ventricle from epicardium to endocardium has been reported to be 140° in rats [49], 135° in guinea pigs [51], 140° in dogs [52], and 106° in mice [51].

Cardiomyocytes and collagen fibrils demonstrated greater rotation and dispersion in the anterior aspects of LVFW and RVFW, compared to the posterior, across four age groups (Tables 3, 4, 5, and 6 and Figs. 7 and 8). This attribute may be associated with the regional differences in mechanical stress and developmental patterning of the ventricles [100]. These structural differences are functional adaptations to the non-uniform mechanical demands placed on different regions of the heart anteriorly and posteriorly, with the anterior wall requiring a more complex and variable fibre arrangement to handle greater rotation and stress (Tables 3, 4, 5, 6, 7 and 8 and Figs. 7 and 8) [101].

Interestingly, RVFW possessed greater cardiomyocyte rotation in the anterior aspects of the younger hearts, whilst LVFW exhibited a greater cardiomyocyte rotation in the anterior adult hearts, compared to the posterior walls (Tables 3, 4, 5, and 6 and Figs. 7 and 8). These differential changes may be driven by the significant differences in regional mechanical stress and pressure loads each chamber and wall experiences during development (fetal/neonatal) and into adulthood [102]. This may also be related to the shift in cardiac workload from being right ventricular dominant in the fetal/neonatal to left ventricular dominant in adulthood. This shift occurs rapidly during the transition to extrauterine life at birth due to significant changes in the circulatory system [103].

## Limitations

While the results reported in this study provide valuable insight into the region-specific and age-dependent microstructural changes in the anterior and posterior aspects of LVFW and RVFW myocardial tissue during ageing from neonatal to adulthood, it is acknowledged that an animal-based laboratory study differs from human physiological reality. The porcine animal model was adopted, as it is a widely accepted animal surrogate model for cardiac-related biomedical research and clinical trials [64–66]. Four age groups of hearts were used in our study, namely 7 days, 14 days, 7 months, and 3 years. Due to the smaller size of the younger hearts, particularly the 7-day-old, it was unfeasible to dissect the appropriate dimensions of the samples from the apical regions to perform the THEF/SHG analysis. Consequently, the apical region is omitted in all age groups because of the unavailability of the 7-day-old data to compare with the adults for coherent analysis and interpretation, spanning from neonatal to adulthood. Nevertheless, the basal and equatorial data of LVFW and RVFW in the anterior and posterior aspects, reported in this study, provide a baseline for the apical data. Female porcine hearts were used to collect, quantify and report the data, consistent and unbiased from the sex dependent changes in microstructure. However, it is acknowledged that there is a sex-dependent cardiac remodelling. Hence, future work is planned to investigate and quantify the sex dependent changes in the microstructure from neonatal to adulthood. While the controlled environment of a laboratory has significant advantages for investigating tissue microstructural characteristics, the need to dissect samples can create artificial boundary conditions, release residual stresses, and the optically clearing of tissues may cause ethanol dehydration artefacts such as shrinkage, which may slightly alter the microstructural arrangement and potentially cause small changes in the rotation and dispersion of cardiomyocytes and collagen fibrils. Common protocols were followed to ensure that the tissues remained hydrated. Regardless of the limitations associated with in vitro microstructural analyses, the approach used here has provided important and novel data sets on heart ageing in the anterior and posterior aspects of both ventricles, from neonatal to adult stages.

## Conclusion

Microstructural analyses revealed prominent microstructural changes in the anterior and posterior aspects of both ventricles across four age groups. The region-specific and age-dependent variations were noted for FA and SM. LVFW possesses greater FA than the RVFW. Whilst RVFW exhibited greater SM than the LVFW. Adult hearts demonstrated smaller FA than those of the young. Variations in cardiomyocyte and collagen fibril rotation and dispersion were reported in both ventricles across regions and age groups. The anterior LVFW and RVFW have greater cardiomyocyte and collagen fibril rotation and dispersion than the posterior. Adult hearts exhibit greater cardiomyocyte and collagen fibril rotation and dispersion than young hearts. RVFW demonstrated greater cardiomyocyte rotation in the younger heart, and the LVFW in the adult. This study provides baseline data that should prove useful to bioengineers, researchers, and mathematicians in developing region-specific and age-dependent constitutive models to enhance the accuracy and bio-fidelity of computational simulations.

## Supplementary Information

Below is the link to the electronic supplementary material.

Supplementary file1 (DOCX 982 KB)

## Data Availability

All relevant data are within the paper.
