# Supplementary material for: Quantifying Regional and Age-Dependent Microstructural Changes in Porcine Ventricles from Neonatal to Adulthood Using DT-MRI and TPEF-SHG Microscopy
Source: Ann Biomed Eng. 2026 Apr 13;54(11):3888–915. doi: 10.1007/s10439-026-04143-4 (PMC13694413; doi:10.1007/s10439-026-04143-4)
Supplement: Supplementary file 1 [file 10439_2026_4143_MOESM1_ESM.docx]

**Supplementary Materials – Figure A.1**

**(a)**

7 Months LVFW - Preferred Fibre Orientation

Frequency

| Preferred Fibre Orientation (degree) Epicardium (Left) to Endocardium (Right) | Cardiomyocytes | Collagen |
| --- | --- | --- |
| -90 | 0 | 0 |
| -80 | 0 | 6 |
| -70 | 0 | 4 |
| -60 | 17 | 0 |
| -50 | 13 | 1 |
| -40 | 5 | 9 |
| -30 | 10 | 11 |
| -20 | 9 | 5 |
| -10 | 47 | 32 |
| 0 | 4 | 5 |
| 10 | 0 | 14 |
| 20 | 111 | 92 |
| 30 | 66 | 21 |
| 40 | 45 | 0 |
| 50 | 0 | 0 |
| 60 | 2 | 6 |
| 70 | 30 | 18 |
| 80 | 3 | 0 |
| 90 | 35 | 2 |
| More | 0 | 0 |

**(b)**

7 Months LVFW - Fibre Dispersion

Frequency

| Fibre Dispersion (degree) Epicardium (Left) to Endocardium (Right) | Cardiomyocytes | Collagen |
| --- | --- | --- |
| 0 | 0 | 0 |
| 5 | 34 | 1 |
| 10 | 292 | 44 |
| 15 | 60 | 67 |
| 20 | 11 | 40 |
| 25 | 6 | 38 |
| 30 | 1 | 22 |
| 35 | 0 | 11 |
| 40 | 0 | 3 |
| 45 | 0 | 0 |
| 50 | 0 | 1 |
| More | 0 | 0 |

**(c)**

7 months LVFW - Fibre Direction

Preferred Orientation (degree)

| Epi - to endocardium (%) | Collagens |
| --- | --- |
| 0 | -11.21 |
| 0.442478 | -12.86 |
| 0.884956 | -14.7 |
| 1.32743 | -24.82 |
| 1.76991 | -24.29 |
| 2.21239 | -18.05 |
| 2.65487 | -17.08 |
| 3.09735 | -19.16 |
| 3.53982 | -19.86 |
| 3.9823 | -21.9 |
| 4.42478 | -20.27 |
| 4.86726 | -18.08 |
| 5.30973 | -15.15 |
| 5.75221 | -16.41 |
| 6.19469 | -19.99 |
| 6.63717 | -21.62 |
| 7.07965 | -18.54 |
| 7.52212 | -19.98 |
| 7.9646 | -15.28 |
| 8.40708 | -16.92 |
| 8.84956 | -11.87 |
| 9.29204 | -12.64 |
| 9.73451 | -12.34 |
| 10.177 | -12.28 |
| 10.6195 | -11.44 |
| 11.0619 | -12.19 |
| 11.5044 | -11.41 |
| 11.9469 | -13.11 |
| 12.3894 | -14.47 |
| 12.8319 | -15.65 |
| 13.2743 | -13.95 |
| 13.7168 | -10.04 |
| 14.1593 | -9.58 |
| 14.6018 | -11.01 |
| 15.0442 | -11.94 |
| 15.4867 | -13.01 |
| 15.9292 | -10.05 |
| 16.3717 | -11.33 |
| 16.8142 | -35.95 |
| 17.2566 | -36.21 |
| 17.6991 | -34.79 |
| 18.1416 | -33.45 |
| 18.5841 | -37.58 |
| 19.0265 | -36.33 |
| 19.469 | -37.05 |
| 19.9115 | -36.75 |
| 20.354 | -35.56 |
| 20.7965 | -38.25 |
| 21.2389 | -39.8 |
| 21.6814 | -41.4 |
| 22.1239 | -43.08 |
| 22.5664 | -45.48 |
| 23.0088 | -40.38 |
| 23.4513 | -43.49 |
| 23.8938 | -43.38 |
| 24.3363 | -45.28 |
| 24.7788 | -48.62 |
| 25.2212 | -45.89 |
| 25.6637 | -53.94 |
| 26.1062 | 16.07 |
| 26.5487 | 15.03 |
| 26.9912 | 16.86 |
| 27.4336 | 18.51 |
| 27.8761 | 18.93 |
| 28.3186 | 19.86 |
| 28.7611 | 18.42 |
| 29.2035 | 17.58 |
| 29.646 | 18.63 |
| 30.0885 | 18.65 |
| 30.531 | 18.78 |
| 30.9735 | 17.05 |
| 31.4159 | 15.31 |
| 31.8584 | 14.37 |
| 32.3009 | 15.89 |
| 32.7434 | 15.82 |
| 33.1858 | 15.89 |
| 33.6283 | 15.42 |
| 34.0708 | 13.63 |
| 34.5133 | 13.64 |
| 34.9558 | 14.69 |
| 35.3982 | 14.03 |
| 35.8407 | 15.44 |
| 36.2832 | 17.27 |
| 36.7257 | 15.62 |
| 37.1681 | 15.92 |
| 37.6106 | 17.07 |
| 38.0531 | 18.19 |
| 38.4956 | 15.9 |
| 38.9381 | 12.74 |
| 39.3805 | 12.28 |
| 39.823 | 17.01 |
| 40.2655 | 6.22 |
| 40.708 | 6.6 |
| 41.1504 | 11.55 |
| 41.5929 | 12.57 |
| 42.0354 | 12.68 |
| 42.4779 | 11.58 |
| 42.9204 | 13.86 |
| 43.3628 | 18.15 |
| 43.8053 | 16.53 |
| 44.2478 | 15.72 |
| 44.6903 | 15.5 |
| 45.1327 | 9.42 |
| 45.5752 | 16.83 |
| 46.0177 | 14.6 |
| 46.4602 | 15.04 |
| 46.9027 | 12.45 |
| 47.3451 | 12.12 |
| 47.7876 | 11.53 |
| 48.2301 | 11.45 |
| 48.6726 | 15.72 |
| 49.115 | 14.76 |
| 49.5575 | 14.95 |
| 50 | 7.96 |
| 50.4425 | 14.57 |
| 50.885 | 12.22 |
| 51.3274 | 10.82 |
| 51.7699 | 12.13 |
| 52.2124 | 84.4 |
| 52.6549 | 83.73 |
| 53.0973 | 83.6 |
| 53.5398 | 77.33 |
| 53.9823 | 81.52 |
| 54.4248 | 91.02 |
| 54.8673 | 85.34 |
| 55.3097 | 79.55 |
| 55.7522 | 81.2 |
| 56.1947 | 77.81 |
| 56.6372 | 80.05 |
| 57.0796 | 77.57 |
| 57.5221 | 84.01 |
| 57.9646 | 6.33 |
| 58.4071 | 5.08 |
| 58.8496 | 0.85 |
| 59.292 | 6.42 |
| 59.7345 | 4.29 |
| 60.177 | 1.52 |
| 60.6195 | 3.27 |
| 61.0619 | 1.23 |
| 61.5044 | 3.45 |
| 61.9469 | 9.93 |
| 62.3894 | 4.79 |
| 62.8319 | 20.45 |
| 63.2743 | 5.53 |
| 63.7168 | 14.62 |
| 64.1593 | 59.92 |
| 64.6018 | 63.57 |
| 65.0442 | 63.29 |
| 65.4867 | 61.55 |
| 65.9292 | 61.29 |
| 66.3717 | 60.07 |
| 66.8142 | 59.13 |
| 67.2566 | 58.23 |
| 67.6991 | 60.3 |
| 68.1416 | 60.56 |
| 68.5841 | 61.44 |
| 69.0265 | 61.53 |
| 69.469 | 61.58 |
| 69.9115 | 62.08 |
| 70.354 | 60.9 |
| 70.7965 | 59.54 |
| 71.2389 | 59.58 |
| 71.6814 | 60.03 |
| 72.1239 | 62.75 |
| 72.5664 | 61.94 |
| 73.0088 | 60.77 |
| 73.4513 | 62.59 |
| 73.8938 | 63.37 |
| 74.3363 | 57.85 |
| 74.7788 | 9.18 |
| 75.2212 | 12.23 |
| 75.6637 | 11.38 |
| 76.1062 | 11.26 |
| 76.5487 | 13.95 |
| 76.9912 | 11.54 |
| 77.4336 | 15.29 |
| 77.8761 | 15.8 |
| 78.3186 | 18.53 |
| 78.7611 | 20.87 |
| 79.2035 | 13.31 |
| 79.646 | 14.58 |
| 80.0885 | 15.83 |
| 80.531 | 11.94 |
| 80.9735 | 13.58 |
| 81.4159 | 14.62 |
| 81.8584 | 15.45 |
| 82.3009 | 15.33 |
| 82.7434 | 14.06 |
| 83.1858 | 15.29 |
| 83.6283 | 15.05 |
| 84.0708 | 15.08 |
| 84.5133 | 15.54 |
| 84.9558 | 15.79 |
| 85.3982 | 16.36 |
| 85.8407 | 14.94 |
| 86.2832 | 16.25 |
| 86.7257 | 17.6 |
| 87.1681 | 18.05 |
| 87.6106 | 18.69 |
| 88.0531 | 17.95 |

|  | Cardiomyocytes |
| --- | --- |
| 0 | -27.81 |
| 0.248139 | -26.57 |
| 0.496278 | -25.41 |
| 0.744417 | -23.15 |
| 0.992556 | -22.72 |
| 1.24069 | -21.05 |
| 1.48883 | -20.58 |
| 1.73697 | -19.89 |
| 1.98511 | -18.44 |
| 2.23325 | -18.24 |
| 2.48139 | -17.64 |
| 2.72953 | -17.38 |
| 2.97767 | -16.63 |
| 3.22581 | -16.63 |
| 3.47395 | -16.48 |
| 3.72208 | -15.52 |
| 3.97022 | -14.75 |
| 4.21836 | -15.23 |
| 4.4665 | -14.66 |
| 4.71464 | -15.15 |
| 4.96278 | -14.88 |
| 5.21092 | -14.93 |
| 5.45906 | -15.7 |
| 5.7072 | -15.04 |
| 5.95533 | -15.86 |
| 6.20347 | -15.61 |
| 6.45161 | -15.89 |
| 6.69975 | -15.14 |
| 6.94789 | -14.49 |
| 7.19603 | -14.56 |
| 7.44417 | -13.07 |
| 7.69231 | -12.87 |
| 7.94045 | -11.52 |
| 8.18859 | -11.77 |
| 8.43672 | -10.09 |
| 8.68486 | -11.76 |
| 8.933 | -11.45 |
| 9.18114 | -10.86 |
| 9.42928 | -11.25 |
| 9.67742 | -10.51 |
| 9.92556 | -11.11 |
| 10.1737 | -10.31 |
| 10.4218 | -9.75 |
| 10.67 | -10.34 |
| 10.9181 | -10.35 |
| 11.1663 | -10.3 |
| 11.4144 | -10.46 |
| 11.6625 | -10.11 |
| 11.9107 | -10.43 |
| 12.1588 | -10.74 |
| 12.4069 | -10.54 |
| 12.6551 | -10.93 |
| 12.9032 | -10.73 |
| 13.1514 | -9.75 |
| 13.3995 | -10.04 |
| 13.6476 | -9.65 |
| 13.8958 | -10.05 |
| 14.1439 | -9.94 |
| 14.3921 | -28.87 |
| 14.6402 | -29.7 |
| 14.8883 | -30.43 |
| 15.1365 | -31.97 |
| 15.3846 | -33.46 |
| 15.6328 | -34.8 |
| 15.8809 | -35.33 |
| 16.129 | -36.58 |
| 16.3772 | -37.31 |
| 16.6253 | -38.74 |
| 16.8734 | -39.39 |
| 17.1216 | -39.98 |
| 17.3697 | -41.44 |
| 17.6179 | -43.17 |
| 17.866 | -44.9 |
| 18.1141 | -47.88 |
| 18.3623 | -49.93 |
| 18.6104 | -51.45 |
| 18.8586 | -52.09 |
| 19.1067 | -54.18 |
| 19.3548 | -55.6 |
| 19.603 | -56.45 |
| 19.8511 | -56.64 |
| 20.0993 | -56.56 |
| 20.3474 | -55.99 |
| 20.5955 | -57.11 |
| 20.8437 | -57.61 |
| 21.0918 | -58.5 |
| 21.34 | -58.37 |
| 21.5881 | -59.21 |
| 21.8362 | -60.93 |
| 22.0844 | -61.46 |
| 22.3325 | -62.68 |
| 22.5806 | -63.92 |
| 22.8288 | -63.44 |
| 23.0769 | -64.41 |
| 23.3251 | -65.1 |
| 23.5732 | -66.17 |
| 23.8213 | -65.34 |
| 24.0695 | -64.05 |
| 24.3176 | -65.93 |
| 24.5658 | -67.42 |
| 24.8139 | -64.95 |
| 25.062 | -65.87 |
| 25.3102 | -66.26 |
| 25.5583 | -65.94 |
| 25.8065 | -65.5 |
| 26.0546 | 18.18 |
| 26.3027 | 18.36 |
| 26.5509 | 18.44 |
| 26.799 | 19.11 |
| 27.0471 | 19.57 |
| 27.2953 | 19.54 |
| 27.5434 | 19.34 |
| 27.7916 | 19.02 |
| 28.0397 | 18.82 |
| 28.2878 | 18.96 |
| 28.536 | 18.8 |
| 28.7841 | 18.56 |
| 29.0323 | 18.38 |
| 29.2804 | 18.19 |
| 29.5285 | 17.87 |
| 29.7767 | 17.45 |
| 30.0248 | 17.16 |
| 30.273 | 17.07 |
| 30.5211 | 16.94 |
| 30.7692 | 16.96 |
| 31.0174 | 16.88 |
| 31.2655 | 16.58 |
| 31.5136 | 16.58 |
| 31.7618 | 16.6 |
| 32.0099 | 16.59 |
| 32.2581 | 16.43 |
| 32.5062 | 16.48 |
| 32.7543 | 16.71 |
| 33.0025 | 16.71 |
| 33.2506 | 16.7 |
| 33.4988 | 16.7 |
| 33.7469 | 16.72 |
| 33.995 | 16.57 |
| 34.2432 | 16.47 |
| 34.4913 | 16.32 |
| 34.7395 | 16.18 |
| 34.9876 | 16.13 |
| 35.2357 | 15.95 |
| 35.4839 | 15.75 |
| 35.732 | 15.54 |
| 35.9801 | 15.46 |
| 36.2283 | 15.23 |
| 36.4764 | 14.77 |
| 36.7246 | 14.32 |
| 36.9727 | 14.17 |
| 37.2208 | 14.06 |
| 37.469 | 14.28 |
| 37.7171 | 14.45 |
| 37.9653 | 14.51 |
| 38.2134 | 14.32 |
| 38.4615 | 14.08 |
| 38.7097 | 14.35 |
| 38.9578 | 14.78 |
| 39.206 | 15.35 |
| 39.4541 | 15.5 |
| 39.7022 | 16.2 |
| 39.9504 | 16.4 |
| 40.1985 | 16.95 |
| 40.4467 | 17.11 |
| 40.6948 | 17.04 |
| 40.9429 | 17.2 |
| 41.1911 | 17.45 |
| 41.4392 | 17.51 |
| 41.6873 | 17.62 |
| 41.9355 | 17.85 |
| 42.1836 | 17.92 |
| 42.4318 | 18.06 |
| 42.6799 | 83.72 |
| 42.928 | 82.43 |
| 43.1762 | 81.95 |
| 43.4243 | 80.78 |
| 43.6725 | 79.59 |
| 43.9206 | 78.92 |
| 44.1687 | 80.01 |
| 44.4169 | 81.04 |
| 44.665 | 81.23 |
| 44.9132 | 81.34 |
| 45.1613 | 81.25 |
| 45.4094 | 81.16 |
| 45.6576 | 81.48 |
| 45.9057 | 82.22 |
| 46.1538 | 83.01 |
| 46.402 | 82.79 |
| 46.6501 | 82.47 |
| 46.8983 | 82.01 |
| 47.1464 | 81.76 |
| 47.3945 | 81.42 |
| 47.6427 | 80.81 |
| 47.8908 | 80.83 |
| 48.139 | 80.92 |
| 48.3871 | 81.17 |
| 48.6352 | 81.34 |
| 48.8834 | 81.07 |
| 49.1315 | 80.68 |
| 49.3797 | 80.46 |

**(d)**

7 months LVFW - Fibre dispersion

Dispersion (degree)

| Epi - to endocardium (%) | Collagens |
| --- | --- |
| 0 | 30.41 |
| 0.442478 | 24.4 |
| 0.884956 | 31.37 |
| 1.32743 | 31.51 |
| 1.76991 | 21.45 |
| 2.21239 | 22.99 |
| 2.65487 | 26.12 |
| 3.09735 | 21.16 |
| 3.53982 | 24.58 |
| 3.9823 | 25.21 |
| 4.42478 | 26.56 |
| 4.86726 | 29.05 |
| 5.30973 | 25.51 |
| 5.75221 | 20 |
| 6.19469 | 23.13 |
| 6.63717 | 23 |
| 7.07965 | 27.83 |
| 7.52212 | 31.53 |
| 7.9646 | 26.13 |
| 8.40708 | 25.1 |
| 8.84956 | 20.71 |
| 9.29204 | 9.76 |
| 9.73451 | 17.29 |
| 10.177 | 7.4 |
| 10.6195 | 17.62 |
| 11.0619 | 11.99 |
| 11.5044 | 12.48 |
| 11.9469 | 9.97 |
| 12.3894 | 17.96 |
| 12.8319 | 19.29 |
| 13.2743 | 21.86 |
| 13.7168 | 18.46 |
| 14.1593 | 26.42 |
| 14.6018 | 26.11 |
| 15.0442 | 22.98 |
| 15.4867 | 8.13 |
| 15.9292 | 17.36 |
| 16.3717 | 35.08 |
| 16.8142 | 22.6 |
| 17.2566 | 23.37 |
| 17.6991 | 28.24 |
| 18.1416 | 28.11 |
| 18.5841 | 26.8 |
| 19.0265 | 26.73 |
| 19.469 | 24.56 |
| 19.9115 | 24.97 |
| 20.354 | 27.14 |
| 20.7965 | 26.34 |
| 21.2389 | 23.8 |
| 21.6814 | 21.68 |
| 22.1239 | 23.16 |
| 22.5664 | 23.95 |
| 23.0088 | 22.44 |
| 23.4513 | 26.97 |
| 23.8938 | 33.89 |
| 24.3363 | 27.44 |
| 24.7788 | 24.18 |
| 25.2212 | 35.12 |
| 25.6637 | 26.59 |
| 26.1062 | 24.41 |
| 26.5487 | 12.11 |
| 26.9912 | 13 |
| 27.4336 | 10.62 |
| 27.8761 | 11.53 |
| 28.3186 | 12 |
| 28.7611 | 12.72 |
| 29.2035 | 13.21 |
| 29.646 | 12.15 |
| 30.0885 | 12.49 |
| 30.531 | 12.27 |
| 30.9735 | 12.97 |
| 31.4159 | 11.1 |
| 31.8584 | 13.27 |
| 32.3009 | 13.48 |
| 32.7434 | 13.87 |
| 33.1858 | 12.22 |
| 33.6283 | 10.69 |
| 34.0708 | 14.7 |
| 34.5133 | 15.14 |
| 34.9558 | 13.54 |
| 35.3982 | 14.9 |
| 35.8407 | 14.28 |
| 36.2832 | 12.97 |
| 36.7257 | 13.4 |
| 37.1681 | 11.21 |
| 37.6106 | 12.23 |
| 38.0531 | 14.3 |
| 38.4956 | 12.38 |
| 38.9381 | 19.2 |
| 39.3805 | 17.99 |
| 39.823 | 17.24 |
| 40.2655 | 8.14 |
| 40.708 | 20.65 |
| 41.1504 | 18.47 |
| 41.5929 | 16.74 |
| 42.0354 | 10.32 |
| 42.4779 | 9.91 |
| 42.9204 | 18.04 |
| 43.3628 | 9.3 |
| 43.8053 | 18.56 |
| 44.2478 | 18.54 |
| 44.6903 | 16.38 |
| 45.1327 | 20.94 |
| 45.5752 | 21.09 |
| 46.0177 | 15.55 |
| 46.4602 | 24.84 |
| 46.9027 | 22.81 |
| 47.3451 | 31.19 |
| 47.7876 | 19.4 |
| 48.2301 | 13.8 |
| 48.6726 | 13.78 |
| 49.115 | 15.13 |
| 49.5575 | 16.78 |
| 50 | 14.47 |
| 50.4425 | 24.8 |
| 50.885 | 16.21 |
| 51.3274 | 21.33 |
| 51.7699 | 19.98 |
| 52.2124 | 10.61 |
| 52.6549 | 13.42 |
| 53.0973 | 9.06 |
| 53.5398 | 20.87 |
| 53.9823 | 21.33 |
| 54.4248 | 13.12 |
| 54.8673 | 15.13 |
| 55.3097 | 11.36 |
| 55.7522 | 14.29 |
| 56.1947 | 10.55 |
| 56.6372 | 10.25 |
| 57.0796 | 18.25 |
| 57.5221 | 22.12 |
| 57.9646 | 19.25 |
| 58.4071 | 24.62 |
| 58.8496 | 24.97 |
| 59.292 | 10.78 |
| 59.7345 | 24.68 |
| 60.177 | 24.87 |
| 60.6195 | 31.9 |
| 61.0619 | 30.19 |
| 61.5044 | 31.69 |
| 61.9469 | 31.16 |
| 62.3894 | 33.65 |
| 62.8319 | 35.42 |
| 63.2743 | 15.28 |
| 63.7168 | 27.54 |
| 64.1593 | 46.34 |
| 64.6018 | 7.83 |
| 65.0442 | 5.26 |
| 65.4867 | 11.96 |
| 65.9292 | 11.95 |
| 66.3717 | 10.59 |
| 66.8142 | 10.12 |
| 67.2566 | 10.63 |
| 67.6991 | 12.6 |
| 68.1416 | 6.95 |
| 68.5841 | 9.42 |
| 69.0265 | 10.1 |
| 69.469 | 9.2 |
| 69.9115 | 9.86 |
| 70.354 | 11 |
| 70.7965 | 10.52 |
| 71.2389 | 4.69 |
| 71.6814 | 6.45 |
| 72.1239 | 5.91 |
| 72.5664 | 8.58 |
| 73.0088 | 8.93 |
| 73.4513 | 6.02 |
| 73.8938 | 7.94 |
| 74.3363 | 6.17 |
| 74.7788 | 13.96 |
| 75.2212 | 17.06 |
| 75.6637 | 11.03 |
| 76.1062 | 19.24 |
| 76.5487 | 8.73 |
| 76.9912 | 10.49 |
| 77.4336 | 18.25 |
| 77.8761 | 19.93 |
| 78.3186 | 11.37 |
| 78.7611 | 7.76 |
| 79.2035 | 9.94 |
| 79.646 | 19.17 |
| 80.0885 | 17.32 |
| 80.531 | 16.69 |
| 80.9735 | 19.57 |
| 81.4159 | 11.06 |
| 81.8584 | 8.54 |
| 82.3009 | 5.09 |
| 82.7434 | 8.27 |
| 83.1858 | 6.45 |
| 83.6283 | 5.83 |
| 84.0708 | 7.7 |
| 84.5133 | 7.66 |
| 84.9558 | 5.67 |
| 85.3982 | 5.93 |
| 85.8407 | 6.05 |
| 86.2832 | 6.84 |
| 86.7257 | 6.57 |
| 87.1681 | 6.22 |
| 87.6106 | 5.69 |
| 88.0531 | 7.67 |

|  | Cardiomyocytes |
| --- | --- |
| 0 | 19.43 |
| 0.248139 | 19.46 |
| 0.496278 | 17.95 |
| 0.744417 | 16.36 |
| 0.992556 | 15.24 |
| 1.24069 | 12.94 |
| 1.48883 | 11.92 |
| 1.73697 | 10.95 |
| 1.98511 | 8.71 |
| 2.23325 | 8.01 |
| 2.48139 | 8.89 |
| 2.72953 | 9.01 |
| 2.97767 | 8.48 |
| 3.22581 | 9.03 |
| 3.47395 | 9.61 |
| 3.72208 | 8.21 |
| 3.97022 | 6.98 |
| 4.21836 | 7.81 |
| 4.4665 | 8.44 |
| 4.71464 | 9.29 |
| 4.96278 | 8.47 |
| 5.21092 | 8.15 |
| 5.45906 | 8.18 |
| 5.7072 | 7.6 |
| 5.95533 | 7.34 |
| 6.20347 | 8.31 |
| 6.45161 | 7.64 |
| 6.69975 | 9.33 |
| 6.94789 | 8.6 |
| 7.19603 | 8.75 |
| 7.44417 | 8.84 |
| 7.69231 | 7.14 |
| 7.94045 | 7.47 |
| 8.18859 | 6.75 |
| 8.43672 | 4.43 |
| 8.68486 | 7.43 |
| 8.933 | 6.99 |
| 9.18114 | 6.47 |
| 9.42928 | 6.24 |
| 9.67742 | 5.62 |
| 9.92556 | 4.99 |
| 10.1737 | 4.14 |
| 10.4218 | 5.77 |
| 10.67 | 4.9 |
| 10.9181 | 3.24 |
| 11.1663 | 3.9 |
| 11.4144 | 7.03 |
| 11.6625 | 4.25 |
| 11.9107 | 4.6 |
| 12.1588 | 5.41 |
| 12.4069 | 4.55 |
| 12.6551 | 4.74 |
| 12.9032 | 4.45 |
| 13.1514 | 3.36 |
| 13.3995 | 5.19 |
| 13.6476 | 3.45 |
| 13.8958 | 3.12 |
| 14.1439 | 4.72 |
| 14.3921 | 11.36 |
| 14.6402 | 13.05 |
| 14.8883 | 13.51 |
| 15.1365 | 13.42 |
| 15.3846 | 13.13 |
| 15.6328 | 13.63 |
| 15.8809 | 13.25 |
| 16.129 | 12.65 |
| 16.3772 | 11.83 |
| 16.6253 | 10.63 |
| 16.8734 | 10.81 |
| 17.1216 | 11.49 |
| 17.3697 | 11.8 |
| 17.6179 | 12.8 |
| 17.866 | 13.34 |
| 18.1141 | 13.14 |
| 18.3623 | 13.1 |
| 18.6104 | 12.28 |
| 18.8586 | 12.71 |
| 19.1067 | 12.33 |
| 19.3548 | 11.47 |
| 19.603 | 10.62 |
| 19.8511 | 10.72 |
| 20.0993 | 12.22 |
| 20.3474 | 12.66 |
| 20.5955 | 11.86 |
| 20.8437 | 13.61 |
| 21.0918 | 13.65 |
| 21.34 | 14.33 |
| 21.5881 | 13.43 |
| 21.8362 | 13.27 |
| 22.0844 | 12.3 |
| 22.3325 | 12.83 |
| 22.5806 | 11.25 |
| 22.8288 | 13.52 |
| 23.0769 | 11.53 |
| 23.3251 | 13.33 |
| 23.5732 | 13.35 |
| 23.8213 | 13.73 |
| 24.0695 | 20.41 |
| 24.3176 | 19.92 |
| 24.5658 | 17.52 |
| 24.8139 | 16.03 |
| 25.062 | 14.71 |
| 25.3102 | 17.45 |
| 25.5583 | 20.3 |
| 25.8065 | 13.98 |
| 26.0546 | 5.93 |
| 26.3027 | 6.05 |
| 26.5509 | 6.3 |
| 26.799 | 5.97 |
| 27.0471 | 5.99 |
| 27.2953 | 6.43 |
| 27.5434 | 6.62 |
| 27.7916 | 6.51 |
| 28.0397 | 6.43 |
| 28.2878 | 6.57 |
| 28.536 | 6.7 |
| 28.7841 | 6.8 |
| 29.0323 | 6.83 |
| 29.2804 | 6.98 |
| 29.5285 | 6.92 |
| 29.7767 | 6.53 |
| 30.0248 | 6.28 |
| 30.273 | 6.25 |
| 30.5211 | 6.41 |
| 30.7692 | 6.6 |
| 31.0174 | 6.75 |
| 31.2655 | 6.87 |
| 31.5136 | 6.53 |
| 31.7618 | 6.12 |
| 32.0099 | 5.86 |
| 32.2581 | 5.64 |
| 32.5062 | 5.66 |
| 32.7543 | 5.62 |
| 33.0025 | 5.51 |
| 33.2506 | 5.56 |
| 33.4988 | 5.55 |
| 33.7469 | 5.57 |
| 33.995 | 5.79 |
| 34.2432 | 5.89 |
| 34.4913 | 6.05 |
| 34.7395 | 6.19 |
| 34.9876 | 6.23 |
| 35.2357 | 6.16 |
| 35.4839 | 6.37 |
| 35.732 | 6.27 |
| 35.9801 | 6.19 |
| 36.2283 | 6.3 |
| 36.4764 | 6.43 |
| 36.7246 | 6.54 |
| 36.9727 | 6.7 |
| 37.2208 | 7.05 |
| 37.469 | 7.14 |
| 37.7171 | 7.15 |
| 37.9653 | 7.1 |
| 38.2134 | 6.74 |
| 38.4615 | 7.01 |
| 38.7097 | 6.86 |
| 38.9578 | 6.71 |
| 39.206 | 6.05 |
| 39.4541 | 5.84 |
| 39.7022 | 5.07 |
| 39.9504 | 4.67 |
| 40.1985 | 4.32 |
| 40.4467 | 3.83 |
| 40.6948 | 3.83 |
| 40.9429 | 3.94 |
| 41.1911 | 3.73 |
| 41.4392 | 3.92 |
| 41.6873 | 3.9 |
| 41.9355 | 3.68 |
| 42.1836 | 3.9 |
| 42.4318 | 3.72 |
| 42.6799 | 8.03 |
| 42.928 | 7.22 |
| 43.1762 | 8.1 |
| 43.4243 | 9.72 |
| 43.6725 | 10.72 |
| 43.9206 | 10.96 |
| 44.1687 | 11.14 |
| 44.4169 | 10.85 |
| 44.665 | 10.31 |
| 44.9132 | 9.84 |
| 45.1613 | 9.26 |
| 45.4094 | 8.88 |
| 45.6576 | 9.64 |
| 45.9057 | 9.8 |
| 46.1538 | 9.98 |
| 46.402 | 9.27 |
| 46.6501 | 8.9 |
| 46.8983 | 8.32 |
| 47.1464 | 8.59 |
| 47.3945 | 8.58 |
| 47.6427 | 8.3 |
| 47.8908 | 7.74 |
| 48.139 | 7.95 |
| 48.3871 | 8.29 |
| 48.6352 | 7.95 |
| 48.8834 | 8.27 |
| 49.1315 | 8.3 |
| 49.3797 | 8.24 |

**(e)**

Average Fibre Angle by Depth

Fibre Angle (degree)

|  | Cardiomyocytes | Collagen fibrils |
| --- | --- | --- |
| Epicardium | -31.52 | -24.36 |
| Mid-wall | 17.03 | 16.88 |
| Endocardium | 39.94 | 28.45 |

**(f)**

Average Dispersion Angle by Depth

Dispersion Angle (degree)

|  | Cardiomyocytes | Collagen fibrils |
| --- | --- | --- |
| Epicardium | 10.37 | 23.49 |
| Mid-wall | 6.27 | 13.1 |
| Endocardium | 7.43 | 14.67 |
